# FBXW7 loss of function promotes esophageal squamous cell carcinoma progression via elevating MAP4 and ERK phosphorylation

**DOI:** 10.1186/s13046-023-02630-3

**Published:** 2023-03-29

**Authors:** Yunzhi Pan, Jing Liu, Yingyin Gao, Yuqing Guo, Changxing Wang, Zhipan Liang, Meiying Wu, Yulan Qian, Yinyan Li, Jingyi Shen, Chenchen Lu, Sai Ma

**Affiliations:** 1 Department of Pharmacy, The Affiliated Infectious Diseases Hospital, Suzhou Medical College of Soochow University, Suzhou, 215007 China; 2 State Key Laboratory of Molecular Oncology, National Cancer Center/National Clinical Research Center for Cancer/Cancer Hospital, Chinese Academy of Medical Sciences and Peking Union Medical College, Beijing, 100021 China; 3 Affiliated Hospital of Integrated Traditional Chinese and Western Medicine, Nanjing University of Traditional Chinese Medicine, Nanjing, 210023 China; 4 Department of Thoracic Surgery, The Affiliated Suzhou Hospital of Nanjing Medical University, Suzhou, 215008 China; 5 Gusu School, Nanjing Medical University, Suzhou, 215008 China; 6 Department of Tuberculosis, The Affiliated Infectious Diseases Hospital, Suzhou Medical College of Soochow University, Suzhou, 215007 China; 7 Department of Pharmacy, The First Affiliated Hospital of Soochow University, Suzhou, 215006 China; 8 Department of Anatomy, Bengbu Medical College, Bengbu, 233030 China; 9 Department of Laboratory, The Affiliated Suzhou Hospital of Nanjing Medical University, Suzhou, 215008 China

**Keywords:** Esophageal squamous cell carcinoma (ESCC), FBXW7 mutation, MAP4, ERK, Phosphorylation

## Abstract

**Background:**

Increasing evidence suggests that FBXW7 has a high frequency of mutations in esophageal squamous cell carcinoma (ESCC). However, the function of FBXW7, especially the mutations, is not clear. This study was designed to investigate the functional significance of FBXW7 loss of function and underlying mechanism in ESCC.

**Methods:**

Immunofluorescence was applied to clarify the localization and main isoform of FBXW7 in ESCC cells. Sanger sequencing were performed to explore mutations of FBXW7 in ESCC tissues. Proliferation, colony, invasion and migration assays were performed to examine the functional roles of FBXW7 in ESCC cells in vitro and in vivo. Real-time RT-PCR, immunoblotting, GST-pulldown, LC–MS/MS and co-immunoprecipitation assay were used to explore the molecular mechanism underlying the actions of FBXW7 functional inactivation in ESCC cells. Immunohistochemical staining were used to explore the expression of FBXW7 and MAP4 in ESCC tissues.

**Results:**

The main FBXW7 isoform in ESCC cells was the β transcript in the cytoplasm. Functional inactivation of FBXW7 led to activation of the MAPK signaling pathway and upregulation of the downstream MMP3 and VEGFA, which enhanced tumor proliferation cell invasion and migration. Among the five mutation forms screened, S327X (X means truncated mutation) had an effect similar to the FBXW7 deficiency and led to the inactivation of FBXW7 in ESCC cells. Three other point mutations, S382F, D400N and R425C, attenuated but did not eliminate FBXW7 function. The other truncating mutation, S598X, which was located outside of the WD40 domain, revealed a tiny attenuation of FBXW7 in ESCC cells. Notably, MAP4 was identified as a potential target of FBXW7. The threonine T521 of MAP4, which was phosphorylated by CHEK1, played a key role in the FBXW7-related degradation system. Immunohistochemical staining indicated that FBXW7 loss of function was associated with tumor stage and shorter survival of patients with ESCC. Univariate and multivariate Cox proportional hazards regression analyses showed that high FBXW7 and low MAP4 was an independent prognostic indicator and prospective longer survival. Moreover, a combination regimen that included MK-8353 to inhibit the phosphorylation of ERK and bevacizumab to inhibit VEGFA produced potent inhibitory effects on the growth of FBXW7 inactivation xenograft tumors in vivo.

**Conclusions:**

This study provided evidence that FBXW7 loss of function promoted ESCC via MAP4 overexpression and ERK phosphorylation, and this novel FBXW7/MAP4/ERK axis may be an efficient target for ESCC treatment.

**Supplementary Information:**

The online version contains supplementary material available at 10.1186/s13046-023-02630-3.

## Background

Esophageal cancer is one of the deadliest malignancies. Esophageal squamous cell carcinoma (ESCC) accounts for 90% of esophageal cancer cases in the Chinese population [1]. ESCC has a poor survival rate because of the difficulty in early diagnosis and lack of treatment. Conventional methods of cancer therapy in ESCC rely on surgery, radiotherapy and chemotherapy. The five-year survival rate of patients remains poor at approximately 20% [2]. Elucidating the molecular mechanism of ESCC is the key to exploring potential biomarkers and candidate drug targets of ESCC, which is important to improve the survival rate and life quality of patients.

FBXW7 (F-box and WD repeat domain-containing 7), also known as hCDC4, is a member of the F-box protein family. FBXW7 is one subunit of SCF ubiquitin ligase, which is an E3-ubiquitin ligase that triggers ubiquitinated proteins to proteasome degradation. FBXW7 is responsible for recognizing and binding to proteins containing the phosphorylated substrates and regulating their degradation [3]. In most of these target proteins exist a conserved sequence of amino acid residues that may be phosphorylated. These amino acid sequences, called CDC4 phospho-degron sequences (CDPs). FBXW7 binding to target proteins requires the substrate sequences to be phosphorylated at specific residues in order to be ubiquitinated and targeted for proteasome degradation. A majority of these sequences were identified as being phosphorylated by GSK3 [4]. Phosphorylated CDPs bind to the WD40 domain of FBXW7, and the target protein is recognized and degraded by the ubiquitination system [5, 6].

FBXW7 is located at 4q31.3 on the human chromosome, an area that is often missing in ESCC [7, 8]. The mutation rate of FBXW7 in ESCC ranges from 4 to 6% [9–12]. The Chinese pan-cancer project in the cBioPortal database showed that the mutation rate of FBXW7 was 6.23% (38/610), which ranked fourth among all tumors (Supplementary Fig. 1). FBXW7 was reduced, and lower expression of FBXW7 predicted a poor prognosis in ESCC [13–15]. High FBXW7 expression could help patients with locally advanced ESCC to have a positively response to chemoradiation therapy [16]. Current research focuses on the up-regulatory mechanism of FBXW7 in ESCC. Competing endogenous RNAs (ceRNAs), such as miR-25, miR-223 and lncRNA-TTN-AS1, are in charge of FBXW7 reduction [15, 17, 18]. However, the implication and regulatory mechanism of FBXW7, especially the mutation forms in ESCC, are not clear.

The present study investigated the potential role and regulatory mechanism of FBXW7, especially mutations in ESCC. We determined that FBXW7 deficiency and certain types of FBXW7 mutations promoted the invasion and migration ability of ESCC cells via ERK phosphorylation, which upregulated VEGFA and MMP3 expression. MAP4 was identified as a novel target of FBXW7 through ubiquitination system at the protein level and high MAP4 expression was associated with FBXW7 loss of function in ESCC. Furthermore, we found high FBXW7 and low MAP4 was an independent prognostic indicator and prospective longer survival. And targeting this FBXW7/MAP4/ERK axis could significantly inhibited tumors induced by FBXW7 inactivation. Taken together, we discovered a novel regulatory FBXW7/MAP4/ERK axis in ESCC that may serve as a potential biomarker and therapeutic target in the future.

## Materials and methods

### Tissue specimens

Fresh ESCC tissues and adjacent non-tumorous tissues were procured from surgical resection specimens collected by the Department of Pathology at the First Affiliated Hospital of Bengbu Medical College, Anhui Province, China. None of the patients received treatment before surgery, and all patients signed informed consent forms for tissue sampling and the isolation and storage of DNA, RNA and protein. Experienced pathologists separated primary tumor regions and morphologically normal operative margin tissues from the same patients, and samples were immediately stored at -80 °C until use. The Ethics Committee of Bengbu Medical College approved the study (LKPZ2021-083).

### Immunohistochemical staining

Tissue microarrays were baked in a 68-degree oven for 120 min. Xylene was added immediately to dewax, and the slices were hydrated in 100%, 85% and 75% gradient ethanol for 5 min each and washed via soaking in a phosphate buffer solution (PBS) for 5 min. The endogenous peroxidase was blocked with 3% hydrogen peroxide for 15 min, and antigens were retrieved with 0.1% sodium citrate buffer for 20 min. Tissue microarray sections were added to a primary anti-FBXW7 antibody (Santa Cruz Bio, USA, 1:100) or anti-MAP4 antibody (Proteintech, China, 1:100) and incubated overnight at 4 degrees (> 8 h). After soaking and washing with PBS, the polymer adjuvant in a PV9000 immunohistochemistry kit (Zhongshan Jinqiao Biotechnology, China) and HRP-labeled goat anti-rat/rabbit IgG polymer were successively added and incubated for 20 min and 30 min at 37 °C, respectively. Diaminobenzidine (DAB) was used for color development, and hematoxylin was used for redyeing. The sections were dehydrated in gradient ethanol for 5 min, soaked in xylene for transparency, and sealed with neutral glue for microscopic examination. The staining intensity of FBXW7 and MAP4 were graded as the scales: 0 (negative), 1 (weak) as low expression and 2 (moderate), 3 (strong) as high expression.

### Cell culture and treatments

The human ESCC cell lines KYSE30, KYSE70, KYSE150, KYSE180, KYSE450 and KYSE510 were purchased from the Chinese Academy of Sciences Cell Bank (Shanghai, China). All ESCC cell lines were cultured in RPMI 1640 medium (Gibco, USA) with 10% (vol/vol) fetal bovine serum (Thermo Scientific, USA), 1% L-glutamine (Gibco) and 1% penicillin–streptomycin sulfate as antibiotics (Thermo Scientific). ESCC cell lines were incubated with with MG132 (Selleck) at 10 nM for 12 h, or with cycloheximide (Sigma, USA) at 100 μg/mL for different lengths of time.

SgRNA and plasmid vectors were transfected into cells using Lipofectamine 2000 (Life Technologies, USA) according to the manufacturer’s instructions. Stable KYSE70 or KYSE180 cells transfected with sgRNA plasmid were selected using puromycin (1 μg/mL, Gibco) or FBXW7 plasmid using G418 (300 μg/mL, Gibco).

### sgRNA, small interfering RNA synthesis and plasmid construction

The sgRNA against human FBXW7 and small interfering RNA against human MAP4 and negative controls were synthesized by IGE BIOTECH (China) (Supplementary Table 1).

Wild-type ORFs for FBXW7 or CHEK1 were PCR-amplified from ESCC cell lines. The ORF of FBXW7 was inserted into pcDNA3.1 cloning vectors by restriction enzyme (NEB, USA). The sequence of the FBXW7 WD40 domain was inserted into pGEX-6P-1 cloning vectors by restriction enzyme (NEB, USA). The ORF of CHEK1 was inserted into pEGFP-C1 cloning vectors by restriction enzyme (NEB, USA). The expression plasmid for MAP4 was cloned in a previous study [19]. Various mutant plasmids were generated using a KO-Plus- Mutagenesis Kit (Toyobo, Japan) (Supplementary Table 2).

### Cell matrigel invasion and migration assays

The invasion and migration assays were performed on Transwell plates (Corning Costar, USA) as described previously [20]. For invasion assay, the Transwell membrane was coated with 300 ng/mL Matrigel (BD Biosciences) before cell inoculation. Then, 1 × 10^5^ KYSE70 cells or 1 × 10^5^ KYSE180 cells were seeded into the top chamber of the Transwell insert. Culture medium was supplemented with 20% FBS, which was used as a chemoattractant in the bottom chamber. After incubation for 60 h at 37 °C. Non-migrated cells in the top chamber were removed with cotton swabs. The cells were fixed in 100% methanol, stained with 0.5% crystal violet (Sigma) and imaged by microscopy.

### Cell proliferation assay

A total of 1 × 10^3^ cells were seeded on 96-well plates with 3 replicates. Cell proliferation was measured by staining with 3-(4,5-dimethylthiazol-2-yl)-2,5-diphenyltetrazolium bromide (MTT, Sigma-Aldrich, USA). Absorbance was measured at 570 nm using an Elx 808 Microplate Reader (BioTek Instruments, USA).

### Colony formation assay

For the colony formation assay, 1 × 10^3^ cells were plated in each well of a 24-well plate (Corning). After 7 days, the cells were immobilized with paraformaldehyde, stained with crystal violet and imaged to analyze the number using light microscopy (Leica, Germany).

### Antibodies and western blot analysis

Total protein was isolated from cells using lysis buffer (Beyotime, China). Immunoblotting was performed with primary antibodies against FBXW7 (Proteintech, China), MAP4 (Proteintech, China), MMP3 (Proteintech, China), VEGFA (Proteintech, China), ERK (CST, USA), phosphorylated ERK (Thr202/Tyr204) (CST, USA), GAPDH (Proteintech, China), His-tag (Proteintech, China), and GFP-tag (Proteintech, China). Secondary antibodies were purchased from CWbiotech (China). The signal was visualized using super enhanced chemiluminescence (ECL) detection reagent (Tanon, China).

### DNA, RNA isolation and PCR

Total DNA was isolated from cells using the Universal Genomic DNA Kit (Cwbiotech). Isolated DNA was used for PCR using a Multiplex PCR MasterMix (Cwbiotech). The primers are listed in Supplementary Table 3.

Total RNA was isolated from cells using the RNApure Tissue & Cell Kit (Cwbiotech). Isolated RNA was used as a template for reverse transcription reactions using a HiFiScript cDNA Synthesis Kit (Cwbiotech).

Quantitative real-time PCR analyses were performed with a PCR mixture containing 1 μmol/L of each primer and SYBR Green Real-Time PCR Master Mixes (Thermo Fisher) using a CFX96 Real-Time PCR Detection System (Bio-Rad, USA). Each sample was run in triplicate, and the mRNA level of GAPHD was used as an internal control. The cycle threshold (Ct) value was normalized to the GAPDH level using the 2^−△△Ct^ method. The primers are listed in Supplementary Table 4.

### Immunofluorescence microscopy

A total of 5 × 10^4^ cells were plated in each well of a 24-well plate (Corning). After 24 h, the cells were fixed, permeabilized, blocked and incubated with primary antibodies against FBXW7 (Proteintech). The bound primary antibody was detected using Cy3–conjugated AffiniPure goat anti-rabbit IgG (H + L) (Proteintech). Fluorescence was detected using confocal microscopy (Leica).

### Co-immunoprecipitation

Total protein was isolated from cells using a non-denaturing lysis buffer (Beyotime). The protein lysate was incubated with FBXW7, MAP4, IgG and Protein G agarose (Thermo Scientific) at 4 °C overnight. The immunoprecipitates were collected via centrifugation and washed with PBS. The mixture was subjected to Western blot.

### Animal experiments

The Committee on the Use of Live Animals of Bengbu Medical College approved all animal experiments (LDKPZ2021-182). For the tumor metastasis assay, six-week-old female NOD/SCID mice (Hfkbio, China) were injected with 1 × 10^6^ KYSE70 cells via the tail vein (*n* = 6 per group). The mice were sacrificed after 6 weeks. Metastastic nodules in lung were fixed in Bouin’s solution (Applygen, China), and the number of metastases was determined. The tumor samples were embedded in paraffin, cut into 5-μm sections, and stained with hematoxylin and eosin (H&E). To evaluate tumor growth, six-week-old male BALB/c mice purchased from Hfkbio were subcutaneously injected with 1 × 10^6^ differently treated KYSE70 cells. To inhibit tumor growth, five-week-old female BALB/c mice were given a subcutaneous injection of 1 × 10^6^ KYSE70 cells. After one week, mice were equally divided into two groups according to the mean tumor volume, which was injected intraperitoneally with PBS (vehicle control) or drug regimen in PBS containing MK-8353 (10 mg/kg, three times per week, Selleck) and bevacizumab (15 mg/kg, three times per week, Genentech, USA) in PBS. Tumor size (length × width^2^ × 0.5) was measured at the indicated time points, and the mice were sacrificed after the tumor volume reached 1.5 cm^3^. The weight and volume of the xenografts were measured and imaged.

### RNA-seq analysis

The FBXW7 plasmid was transfected into cells and cultured for 48 h. Total RNA was isolated using the RNApure Tissue and Cell Kit, and the RNA was sequenced via the 150 bp paired method. Hisat2, samtools, and htseq-count packages were used to align 150 bp paired-end reads with hg19 (UCSC). The expression of genes and transcripts was quantified using cufflinks tools.

### Bioinformatics analysis

To analyze the expression levels of FBXW7 and MAP4 in esophageal squamous cell carcinoma, gene expression profiling datasets were obtained from The Cancer Genome Atlas (TCGA) and GEO database (GSE53622 and GSE53624).

### Statistical analysis

All statistical analyses were performed using SPSS 22.0 (SPSS Inc.). The experimental results were statistically evaluated using Student’s t test for two groups and ANOVA for more than two groups. *P* < 0.05 was considered statistically significant. The Kaplan–Meier (KM) survival curve was drawn using the R package "survival" to evaluate the correlation between pathological parameters and prognosis. Univariate and multivariate Cox regression analyses were used to test whether pathological parameters were independent prognostic predictors.

## Results

### FBXW7β is the main form in ESCC cells and is almost mutated in the WD40 domain

**Fig. 1:**
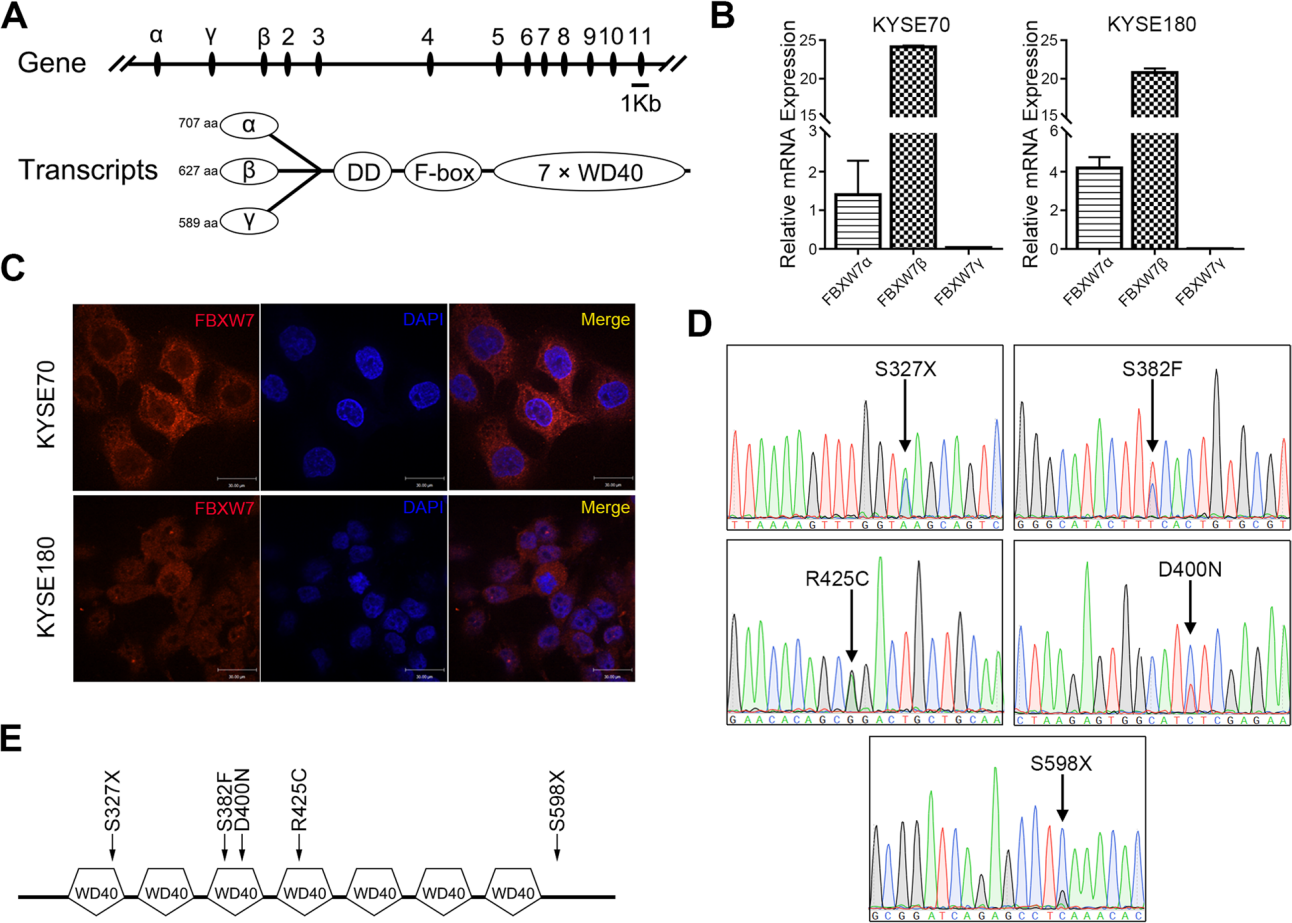
FBXW7β is the main form in ESCC cells and is almost mutated in the WD40 domain. Schematic diagram of the structure of different FBXW7 isoforms. **A** mRNA levels of different FBXW7 isoforms in ESCC cells. **B** Immunofluorescence of FBXW7 in ESCC cells. **C** The results of FBXW7 mutation site using Sanger sequencing in ESCC tissues. **D** Schematic diagram of the location of mutation sites in the FBXW7 WD40 domain. **E** Schematic diagram of the location of mutation sites in the FBXW7 WD40 domain

FBXW7 has three transcripts in cells, FBXW7α, FBXW7β and FBXW7γ, which differ in their 5’-UTR and N-terminal coding regions. FBXW7α is primarily located in the nucleoplasm, FBXW7β is primarily located in the cytoplasm, and FBXW7γ is primarily located in the nucleolus [21]. We first examined the main form of FBXW7 in ESCC cells. As shown in Fig. 1A, the difference between the three transcripts is the first exon. Therefore, we designed three targeted primers for the detection of the three transcripts using qPCR. The results indicated that FBXW7β was the main form in ESCC cells (Fig. 1B). A general antibody against FBXW7 was used for further authentication. Immunofluorescence results showed that FBXW7 was primarily located in the cytoplasm (Fig. 1C).

We explored the mutations of FBXW7 with Sanger sequencing. Mutations were found in 5 of the 120 cases (Fig. 1D). As shown in Fig. 1E, 4/5 mutations were located in the WD40 domain of FBXW7, which indicated that this domain was significant in the function of FBXW7. Notably, among the five mutation forms, we found that S327X was in the first repeat of the WD40 domain. S382F and D400N were situated in the third repeat of the WD40 domain. Mutation R425C was in the fourth repeat of the WD40 domain. The other mutation, S598X, was located in the C-terminus of FBXW7. The mutation sites and sequences were exhibited in Supplementary Fig. 2.

### Loss of FBXW7 promotes the tumorigenic ability of ESCC

**Fig. 2:**
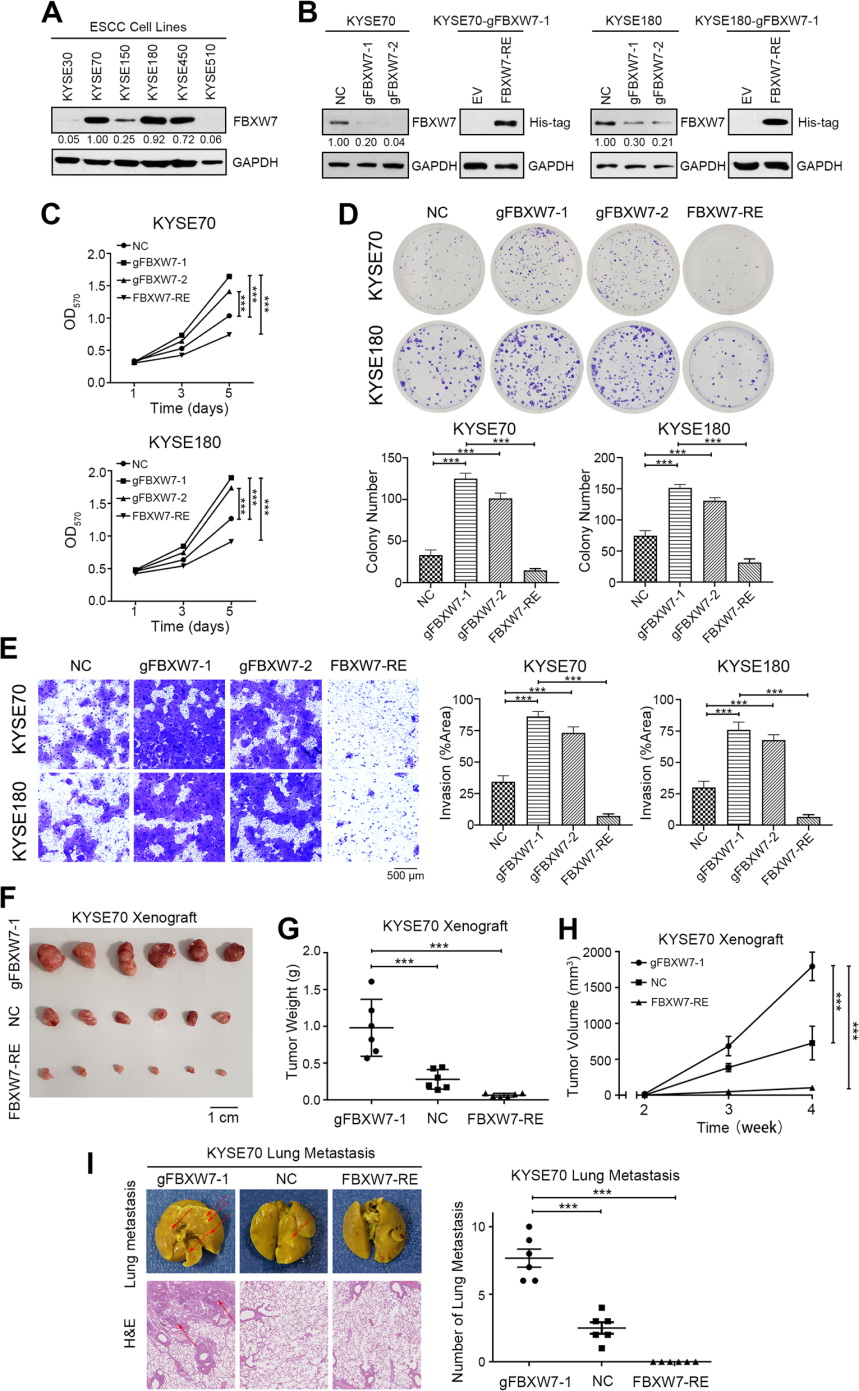
Loss of FBXW7 promotes the tumorigenic ability of ESCC. **A** Western blot analysis of FBXW7 expression in ESCC cell lines. **B** Western blot analysis of FBXW7 expression in KYSE70 and KYSE180 cells after sgRNA and transfected with plasmid in gFBXW7-1 cells. **C** Cell proliferation assay after FBXW7 knockout (gFBXW7-1 or gFBXW7-2) or rescued with FBXW7 plasmid or EV (empty vector) in gFBXW7-1 cells (FBXW7-RE). **D** Representative images and statistical plots of colony growth in KYSE70 and KYSE180 cells after FBXW7 knockout or rescued with FBXW7 plasmid in gFBXW7-1 cells. **E** Representative images and statistical plots of invasion assays in KYSE70 and KYSE180 cells after FBXW7 knockout or rescued with FBXW7 plasmid in gFBXW7-1 cells. **F** Representative images of tumors from the indicated group. **G** The weights of tumors from the indicated groups. **H** Tumor growth curve of tumors from the indicated groups. **I** Representative images and statistical plots of the lung metastatic nodules and the results of H&E staining are shown from the indicated group. Mean ± SEM are shown. *, *p* < 0.05, **, *p* < 0.01, ***, *p* < 0.001

Firstly, we detected the expression of FBXW7 in six esophageal cancer cell lines by Western blot and found that FBXW7 presented strong expression in less malignant cell lines such as KYSE70 and KYSE180, but lower in the higher malignant KYSE30, KYSE150 and KYSE510 (Fig. 2A and Supplementary Fig. 3A). To clarify the biological functions of the FBXW7 mutation in ESCC cells, CRISPR was used to knock out FBXW7, and a FBXW7 plasmid was used to rescue FBXW7 in FBXW7-KO cells (Fig. 2B and Supplementary Fig. 3B). We found that the proliferation of ESCC cells increased after FBXW7 knockout (gFBXW7-1 and gFBXW7-2) but decreased in FBXW7 recovered after overexpression of FBXW7 in gFBXW7-1 cells (FBXW7-RE, Fig. 2C). The colony forming capacity was also enhanced after knockout of FBXW7 (Fig. 2D). Then we investigated the role of FBXW7 in regulating the invasion and migration ability of ESCC cells. FBXW7-KO cells exhibited remarkably strengthened invasion and migration ability compared to the negative control (Fig. 2E and Supplementary Fig. 4). Xenografts of FBXW7-OE cells were significantly inhibited compared to the other xenografts. The gFBXW7-1 group showed the most tumorigenic phenotype (Fig. 2F-H). And FBXW7-RE tumors were significantly inhibited as compared with the others (Fig. 2F-H). Moreover, we performed pulmonary metastasis assay and found the number of lung metastases in the gFBXW7-1 group was much higher than that in the NC group. Cancer cells in the FBXW7-RE group lost the ability to metastasize to the lung. H&E staining of paraffin-embedded lung tissues further confirmed that (Fig. 2I). Collectively, these data demonstrated that FBXW7 knockout promoted the proliferation, invasion and migration ability of ESCC cells.

### Different FBXW7 mutations have different functions in ESCC cells

**Fig. 3:**
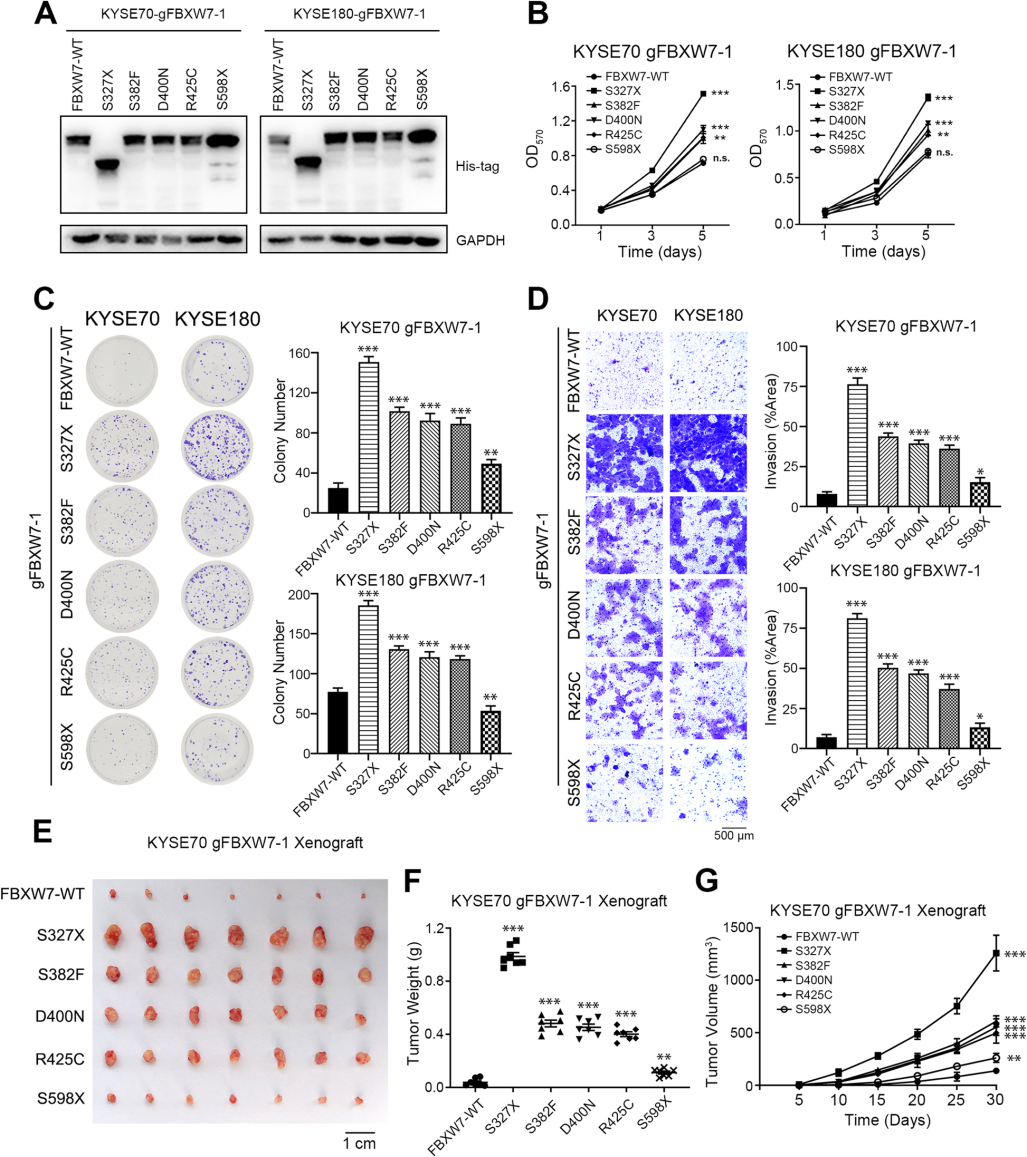
Different FBXW7 mutations have different functions in ESCC cells. **A** Western blot analysis of different FBXW7 plasmid transfected in FBXW7-KO cells (gFBXW7-1). **B** The cell proliferation assay of KYSE70 and KYSE180 cells with reversion of different FBXW7 plasmids transfected in FBXW7-KO cells. **C** Representative images and statistical plots of colony growth in KYSE70 and KYSE180 cells with reversion of different FBXW7 plasmids transfected in FBXW7-KO cells. **D** Representative images and statistical plots of invasion assays in KYSE70 and KYSE180 cells with reversion of different FBXW7 plasmids transfected in FBXW7-KO cells. **E** Representative images of tumors from the indicated groups. **F** The weights of tumors from the indicated groups. **G** Tumor growth curve of tumors from the indicated groups. Means ± SEM are shown. *, *p* < 0.05, **, *p* < 0.01, ***, *p < *0.001; n.s., not significant

To examine the function of FBXW7 mutations, an FBXW7 plasmid containing different mutations was transfected into FBXW7 KO cells (gFBXW7-1, Fig. 3A). S327X mutation demonstrated the strongest proliferative ability. The S382F, D400N and R425C mutations had a less influential effect in ESCC cells compared to the S327X form. However, there was almost no difference between S598X and WT in the proliferation and colony formation assays (Fig. 3B and C). The results were similar in the invasion and migration assays (Fig. 3D and Supplementary Fig. 5). Xenografts of S327X mutation cells showed the most tumorigenic phenotype, followed by the S382F, D400N and R425C mutations. The degree of malignancy of S598X and WT was minimal (Fig. 3E-G). In conclusion, these results showed that not all mutations of FBXW7 were tumor-promoting. Mutation closer to the N-terminus of the WD domain had stronger tumor-promoting effect.

### FBXW7 loss of function accelerates tumor via ERK phosphorylation

**Fig. 4:**
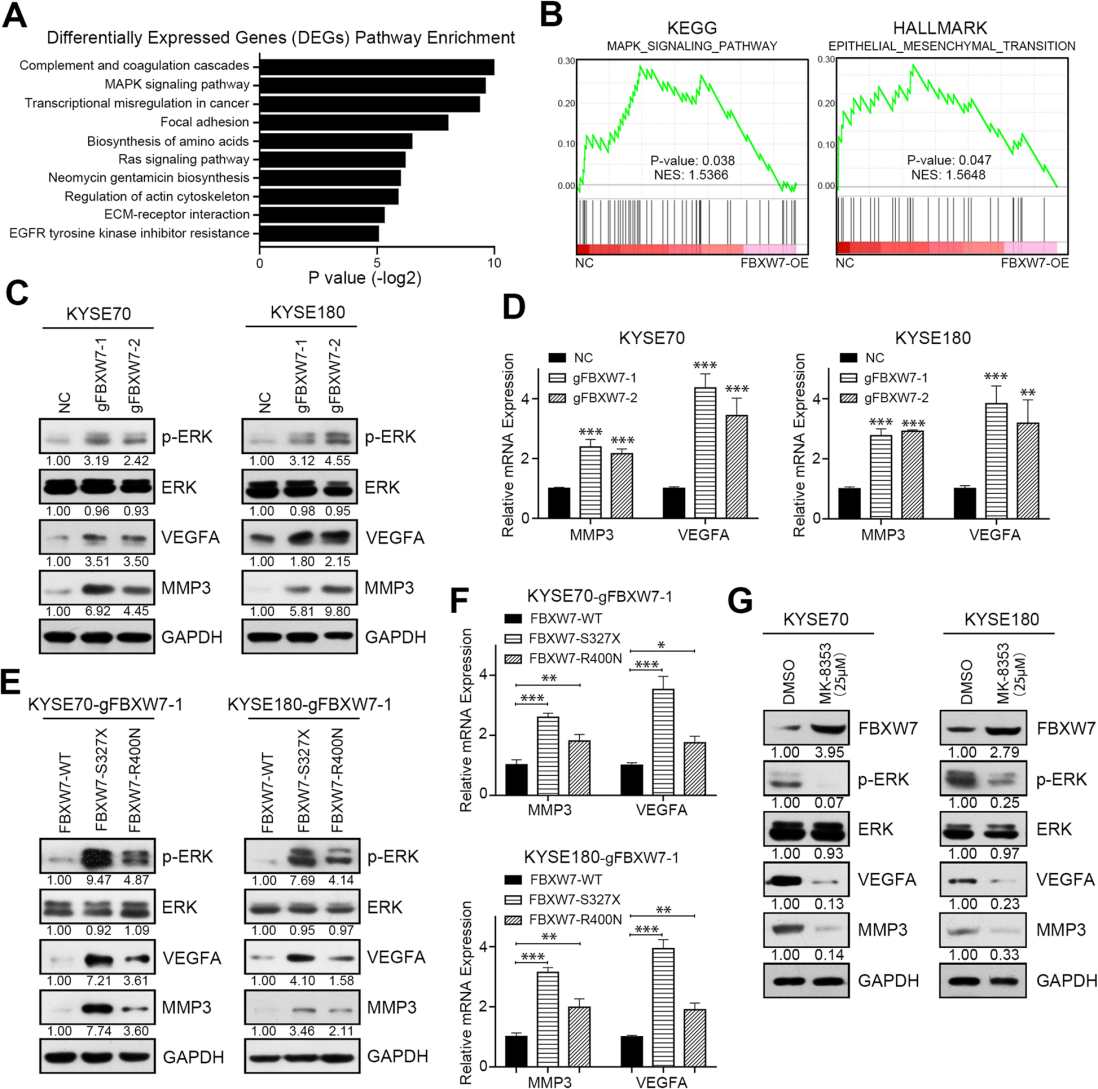
FBXW7 loss of function accelerates tumor progression via ERK phosphorylation.** A** Top 10 enriched pathways of differentially expressed genes following FBXW7 overexpression in KYSE70 cells. **B** GSEA analysis of RNA-seq upon negative control and FBXW7 overexpression in KYSE70 cells. **C** Western blot analysis of p-ERK, ERK, VEGFA, MMP3 and GAPDH in negative control and FBXW7 knockout KYSE70 and KYSE180 cells. **D** Real-time analysis of MMP3 and VEGFA mRNA in negative control and FBXW7 knockout KYSE70 and KYSE180 cells. **E** Western blot analysis of p-ERK, ERK, VEGFA, MMP3 and GAPDH in KYSE70 and KYSE180 cells in which FBXW7 was knocked out and transfected with different FBXW7 plasmids. **F** Real-time analysis of MMP3 and VEGFA mRNA in KYSE70 and KYSE180 cells in which FBXW7 was knocked out and transfected with different FBXW7 plasmids. **G** Western blot analysis of FBXW7, p-ERK, ERK, VEGFA, MMP3 and GAPDH in KYSE70 and KYSE180 cells treated with DMSO or the ERK inhibitor MK-8353. Means ± SEM are shown. *, *p* < 0.05, **, *p* < 0.01, ***, *p* < 0.001

To examine the molecular mechanism of FBXW7 regulation of the malignant characteristics of ESCC, RNA-seq was used to search for potential FBXW7 targets and signaling pathways. After FBXW7 was overexpressed, the MAPK and Ras signaling pathways were significantly enriched (Fig. 4A and Supplementary Table 5). Differentially expressed genes (DEGs) after FBXW7-OE were used to perform unbiased GSEA. Notably, the MAPK pathway in KEGG analysis ranked highest, and the EMT pathway ranked highest in the hallmark analysis, which indicated that the presence of FBXW7 played a role in inhibiting EKR phosphorylation and EMT phenotype (Fig. 4B). Western blot assays showed that ERK phosphorylation was activated after FBXW7 knockout (Fig. 4C). Therefore, we screened the DEGs involved in the MAPK and EMT pathways. VEGFA and MMP3 exhibited increased mRNA and protein levels after FBXW7 knockout (Fig. 4C-D and Supplementary Fig. 6A). However, the presence of the FBXW7 mutation did not suppress the activation of ERK compared with the wild-type (Fig. 4E and Supplementary Fig. 6B). The S327X mutation induced more ERK phosphorylation and mRNA and protein levels of VEGFA and MMP3 and the facilitation was weakened in D400N mutation contrast to the wild-type (Fig. 4E and F). To further clarify the regulatory effect of FBXW7 on VEGFA and MMP3, we used a novel ERK inhibitor with clinical potential, MK-8353, to restrain the activation of ERK phosphorylation [22, 23]. The existence of VEGFA and MMP3 was abolished after the inhibition of ERK phosphorylation (Fig. 4G and Supplementary Fig. 6C). In addition, decreased ERK phosphorylation also restored FBXW7 expression in ESCC cells, which was confirmed in other studies (Fig. 4G and Supplementary Fig. 6C).

### MAP4 is a novel target of FBXW7 via the phosphorylated threonine T521 modified by CHEK1 in ESCC

**Fig. 5:**
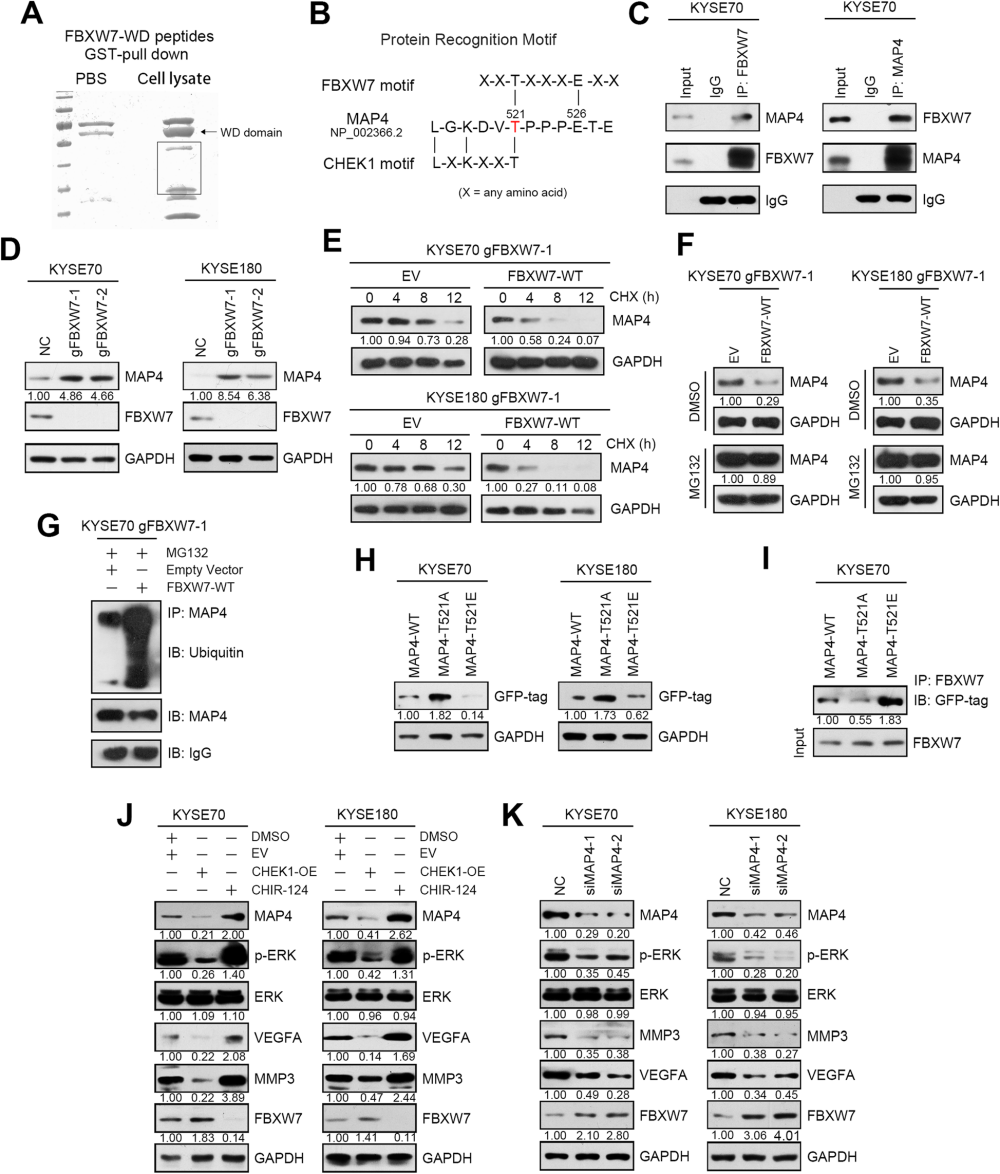
MAP4 is a novel target of FBXW7 via the phosphorylated threonine T521 modified by CHEK1 in ESCC. **A** Image of SDS-PAGE gel includes protein complex that is GST-FBXW7 incubated with cell lysate or PBS. **B** Schematic diagram of the typical FBXW7 recognition motif, the potential protein sequence in MAP4 and CHEK1 phosphorylation motif. **C** Immunoprecipitation analysis of the interaction between FBXW7 and MAP4 in KYSE70 cells. **D** Western blot analysis of MAP4, FBXW7 and GAPDH in negative control and FBXW7 knockout KYSE70 and KYSE180 cells. **E** FBXW7-KO cells (gFBXW7-1) transfected with FBXW7 plasmid or EV (empty vector) were treated with cycloheximide (CHX) for different lengths of time and MAP4 protein levels were examined by Western blot. GAPDH was used as the loading control. **F** FBXW7-KO cells transfected with FBXW7 plasmid or EV were treated with DMSO or the proteasomal inhibitor MG132 and MAP4 protein levels were examined by Western blot. GAPDH was used as the loading control. **G** FBXW7-KO cells transfected with FBXW7 plasmid or EV were treated with the proteasomal inhibitor MG132 and subjected to an immunoprecipitation assay with MAP4 antibody. The level of ubiquitinated MAP4 was detected via Western blot with a ubiquitin antibody. **H** Western blot analysis of MAP4 and GAPDH expression in KYSE70 and KYSE180 cells transfected with different MAP4 plasmids. **I** Immunoprecipitation analysis of the interaction between FBXW7 and different MAP4 plasmids in KYSE70 cells. **J** Western blot analysis of p-ERK, ERK, VEGFA, MMP3, FBXW7, MAP4 and GAPDH in the negative control, CHEK1-overexpressing KYSE70 and KYSE180 cells treated with the CHEK1 inhibitor CHIR-124. **K** Western blot analysis of MAP4, p-ERK, ERK, MMP3, VEGFA, FBXW7 and GAPDH in negative control and MAP4 knockdown KYSE70 and KYSE180 cells

To further understand the regulation of FBXW7 in ESCC cells, we performed a MASS screening assay using GST pulldown to identify novel FBXW7 binding partners (Fig. 5A and Supplementary Table 6). Almost all FBXW7 target proteins possess a sequence named CDPs, and the proteins within CDPs should be phosphorylated then recognized by FBXW7. The predicted proteins were further analyzed using CDP sequences. MAP4 was identified as a potential target of FBXW7 (Fig. 5B). The interaction of FBXW7 and MAP4 was further confirmed via immunoprecipitation using cell lysates obtained from ESCC cells (Fig. 5C).

We found MAP4 was upregulated after FBXW7 knockout, indicating that MAP4 is a downstream of FBXW7 (Fig. 5D and Supplementary Fig. 7A). Sequencing data confirmed that the RNA level of MAP4 was basically stable after the expression of FBXW7 was elevated and the cycloheximide experiment showed that FBXW7 overexpression could accelerate the attenuation of MAP4 protein, indicating that the regulation of MAP4 by FBXW7 occurred at the protein level (Fig. 5E, Supplementary Fig. 7B and Supplementary Table 5). FBXW7 is a member of the ubiquitination complex, so we explored the regulatory mechanism of FBXW7 on MAP4 ubiquitination. Treatment of cells with proteasome inhibitor MG132 could restore MAP4 protein levels which were decreased due to FBXW7 overexpression (Fig. 5F and Supplementary Fig. 7C). And the specific ubiquitination co-immunoprecipitation experiment further that MAP4 was regulated by FBXW7 through ubiquitination system (Fig. 5G).

Then we attempted to identify this MAP4 phosphorylation motif in the PHOSIDA database. The predicted motifs were further analyzed in the PhosphoSitePlus database. The phosphorylation motif L-G-K-D-V-T was identified as a phosphorylation motif by CHEK1 (Fig. 5B). Threonine T521 of MAP4 may be a potential phosphorylation residue involved in FBXW7 recognition. Site-directed mutagenesis of MAP4 from T521T to T521A or T521E with WT plasmid was transfected into ESCC cells. The results showed that the T521A isoform increased, and T521E was significantly reduced compared to MAP4-WT (Fig. 5H and Supplementary Fig. 7D). The interaction between MAP4 and FXBW7 nearly disappeared after transfection with MAP4-T521A and was significantly enhanced with MAP4-T521E via immunoprecipitation (Fig. 5I and Supplementary Fig. 7E). We used CHEK1 inhibitor CHIR-124 to inhibit MAP4 phosphorylation [24]. The expression of MAP4 was increased after CHIR-124 treatment but decreased following CHEK1 overexpression (Fig. 5J and Supplementary Fig. 7F). In conclusion, MAP4 threonine T521 is the key amino acid of phosphorylation modified by CHEK1 and is recognized by FBXW7.

Our previous study demonstrated that MAP4 was an oncogene in ESCC. MAP4 is a protein-binding partner of ERK that promotes ESCC cell invasion and migration by activating the ERK-VEGFA pathway [19]. In this study, we found ERK phosphorylation, VEGFA and MMP3 were also inhibited after MAP4 knockdown, further confirmed that MAP4 played an important role in promoting esophageal cancer (Fig. 5K and Supplementary Fig. 7G).

### High FBXW7 expression with low MAP4 is a novel prognostic marker combination for ESCC

**Fig. 6:**
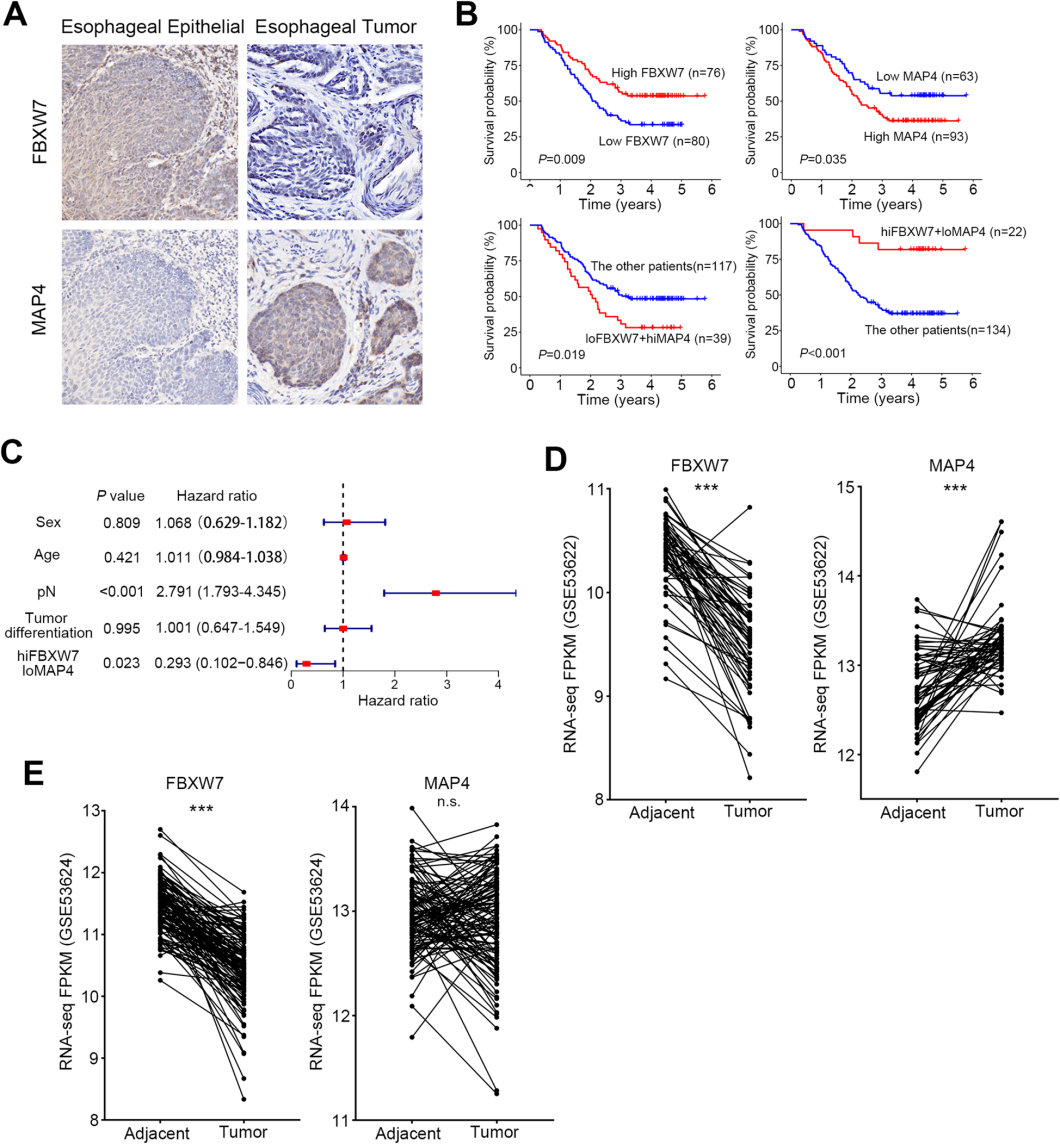
High FBXW7 expression with low MAP4 is a novel prognostic marker combination for ESCC. **A** Representative immunohistochemical photos of FBXW7 and MAP4 expression in tumor samples and the corresponding normal epithelia. **B** Kaplan–Meier (KM) survival curves of the indicated groups (loFBXW7 means FBXW7 low expression and hiMAP4 means MAP4 high expression). **C** Multivariate Cox proportional hazards regression analysis of different parameters. **D** The RNA-seq FPKM of FBXW7 and MAP4 in GSE53622 RNA-seq data. **E** The RNA-seq FPKM of FBXW7 and MAP4 in GSE53624 RNA-seq data. ***, *p* < 0.001; n.s., not significant

We analyzed FBXW7 and MAP4 expression using an immunohistochemical (IHC) technique and a tissue microarray. Low expression of FBXW7 was detected in 80 of the 156 tumors (50.3%, Fig. 6A, Supplementary Table 7). FBXW7 loss of function was significantly associated with tumor size (pT, *P* < 0.001, Supplementary Table 7). Kaplan–Meier analysis of 156 cases showed that patients with low FBXW7 expression had significantly poorer overall survival than patients with strong FBXW7 staining (*P* = 0.009, Fig. 6B). High MAP4 was detected in 93 tumors of 156 patients (59.6%, Fig. 6A, Supplementary Table 8). The high expression of MAP4 was significantly associated with tumor differentiation (*P* = 0.034), tumor size (pT, *P* = 0.005) and lymph node metastasis (pN, *P* = 0.012, Supplementary Table 8). Kaplan–Meier analysis of 156 cases showed that patients with high MAP4 expression had significantly poorer overall survival than patients with low MAP4 staining (*P* = 0.035, Fig. 6B), which is consistent with a previous study [19].

To examine the clinical implications of the above findings, we investigated the relationship between FBXW7 and MAP4 expression in 156 ESCC tissues. We observed that 39 (25.0%) tumors had more intense staining of MAP4 and lower FBXW7 expression, and 22 (14.1%) tumors had higher FBXW7 expression with lower MAP4 expression. Statistical analysis showed a negative correlation between FBXW7 and MAP4 expression in the ESCC tissues tested (*P* = 0.005, Supplementary Table 9). And the ESCC tissues of low FBXW7 along with high MAP4 were significantly associated with tumor differentiation (*P* = 0.01), tumor size (pT, *P* = 0.002) and lymph node metastasis (pN, *P* = 0.029, Supplementary Table 10). Kaplan–Meier analysis of 156 cases showed that patients with low FBXW7 and high MAP4 expression had significantly poorer overall survival than the other patients (*P* = 0.019, Fig. 6B). Notably, patients with high FBXW7 and low MAP4 expression had a better prognosis (*P* < 0.001, Fig. 6B).

To further confirm that FBXW7 and MAP4 have prognostic impacts on survival, we included clinicopathological parameters (sex, age, tumor differentiation, lymph node metastasis) and the expression of FBXW7 and MAP4 to perform univariate and multivariate Cox proportional hazards regression analyses. The results showed that the survival of patients with high FBXW7 and low MAP4 expression was significantly higher than the other patients, which was a potential protective marker and independent prognostic factor (*P* = 0.023, Fig. 6C and Supplementary Table 11).

We examined the changes and correlations of the above molecules in ESCC based on the transcription level. We analyzed the RNA expression data and correlation of FBXW7 and MAP4 from the TCGA and GEO databases. The mRNA levels of FBXW7 in GSE53622 were significantly decreased, and MAP4 was significantly increased in tumor tissues compared to adjacent tissues (Fig. 6D-E). The mRNA levels of FBXW7 in GSE53622 were significantly decreased in tumor tissues compared to adjacent tissues, but there was no obvious trend in MAP4 between tumor and adjacent tissues (Fig. 6D-E). The expression of FBXW7 in the TCGA database was lower, and the expression of MAP4 was higher in tumor tissues compared to adjacent tissues, but the difference was not significant (Supplementary Fig. 8A-B). There was no correlation between the mRNA levels of MAP4 and FBXW7 in any of the three databases (Supplementary Fig. 9A-C).

### Blockade of the MAP4/FBXW7/ERK axis suppresses esophageal squamous cell carcinoma cell growth

**Fig. 7:**
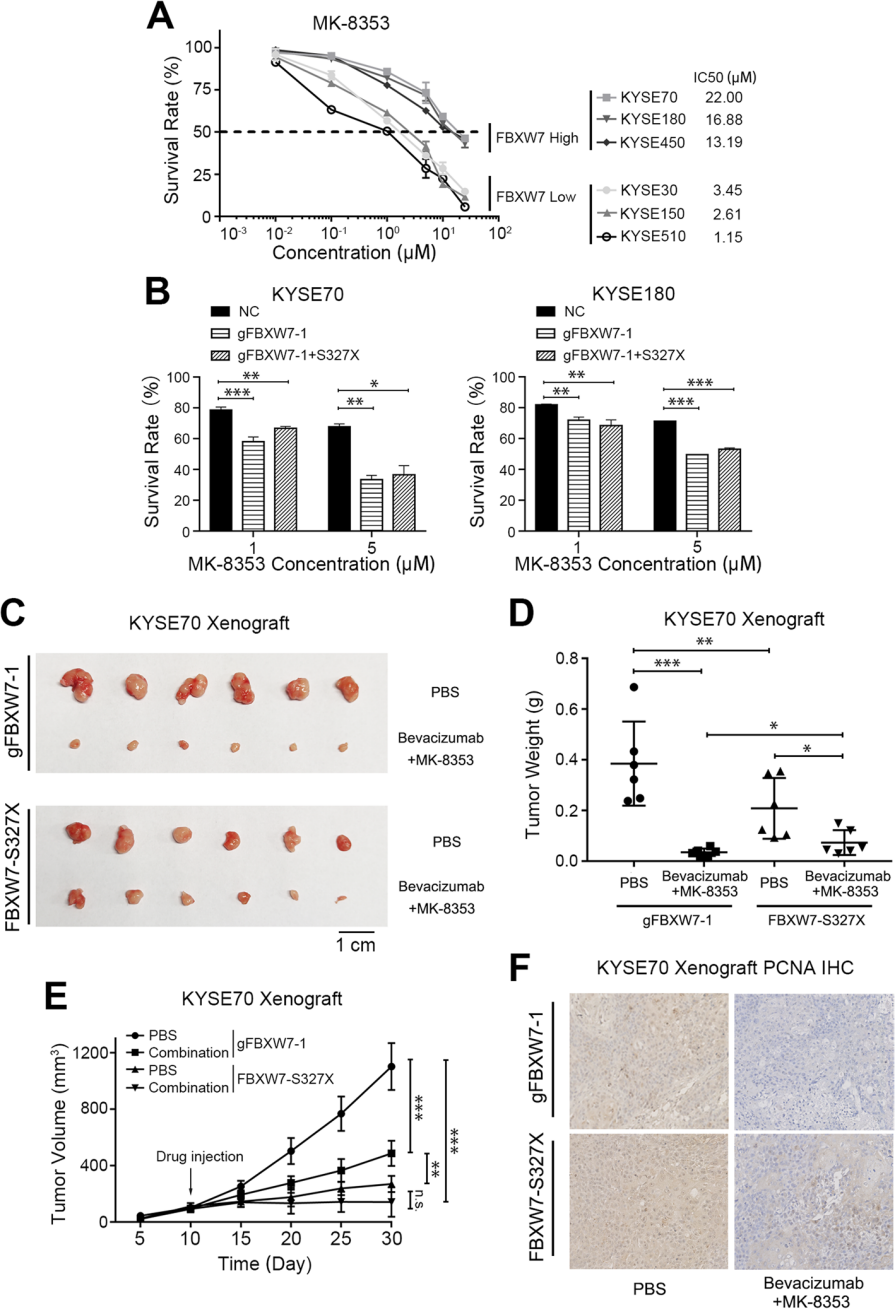
Blockade of the FBXW7/MAP4/ERK axis suppresses esophageal squamous cell carcinoma cell growth. **A** Short-term cell viability assay measuring the IC50s of MK-8353 in different ESCC cell lines. **B** The survival rate of FBXW7 wild-type cells, FBXW7-KO cells (gFBXW7-1) or FBXW7-KO cells transfected with FBXW7-S327X which were treated with different concentration of MK-8353. **C** Representative images of tumors from the indicated groups. **D** The weights of tumors from the indicated groups. **E** Tumor growth curve of tumors from the indicated groups. **F** Immunohistochemical photos of PCNA expression in tumor samples from the indicated groups. Means ± SEM are shown. *, *p* < 0.05, **, *p* < 0.01, ***, *p* < 0.001

The present study confirmed that loss of FBXW7 function effectively induced the proliferation and metastasis of cancer cells via MAP4-ERK-VEGFA. According to this axis, we examined the therapeutic effect in FBXW7-abnormal tumors. We treated ESCC cells with MK-8353 and found that the IC50 value was dependent on the expression of FBXW7 (Fig. 7A). When we changed the state of FBXW7, such as knockout or transfection mutants, the inhibitory effect of MK-8353 also changed (Fig. 7B). MK-8353 exerted better effects when FBXW7 exhibited a loss of function.

Based on these results, we designed a combination regimen to target the different states of FBXW7 abnormalities to evaluate antitumor efficacy in vivo*.* MK-8353 to block the phosphorylation of ERK, we also used bevacizumab to inhibit VEGFA. We mixed the above two factors together to create a novel cocktail therapy. The results showed that the growth of the xenografts was significantly inhibited in the FBXW7-KO groups and slightly weaker in the mutant group (Fig. 7C-F and Supplementary Fig. 10).

## Discussion

Large-scale next-generation sequencing recently has identified many high-frequency mutations in esophageal cancer, but functional research on these mutations is lacking. We investigated the mechanism of FBXW7 loss of function in ESCC and found a novel regulatory FBXW7/MAP4/ERK axis in ESCC. FBXW7 was decreased and mutated in ESCC, and elevated MAP4 increased activation of the ERK pathway, which enhanced the characteristics of cancer cells.

Literatures on tumor-related mutations primarily focuses on comparisons between tumor tissues and tumor adjacent tissues. In recent years, more and more studies have focused on the detection of mutations in normal tissues, disclosed mutations are also present in normal aged tissues [25]. Comparing esophageal epithelial tissues from a healthy population with tumor tissues from esophageal squamous cell carcinoma patients showed a large number of mutations in normal tissue that were previously thought to be tumor-related, such as *TP53* and *FAT1 *[26]. These mutations also occur in normal tissue, but at a lower frequency. Even more, the mutation rate of *NOTCH1* in esophagus is higher than that in tumor tissues. Only *NFE2L2*, *CDKN2A*, and *FBXW7* were rarely mutated in normal samples than cancer samples [27, 28]. These studies reveal that the sequencing results may be biased in real situations, and whether the mutation leads to tumor development must be verified in multiple perspectives and experiments.

In esophageal cancer, current studies focus on the regulation of FBXW7 expression [15, 17, 18, 29]. The function and regulatory mechanism of FBXW7 has rarely been studied in ESCC. There are three transcripts of FBXW7, and we found that FBXW7 was primarily expressed in the cytoplasm in ESCC cells. Knockout of FBXW facilitated the tumorigenic ability of ESCC. Loss of FBXW7 promoted tumor proliferation, invasion and migration ability via VEGFA and MMP3 activation through ERK phosphorylation. The regulation between FBXW7 and VEGFA was also confirmed in nasopharyngeal carcinoma [30]. Besides, the present study further focused on the function of mutated FBXW7 in ESCC. We screened FBXW7 mutations in eight FBXW7 cell lines and found that FBXW7 was wild-type in all of these cells lines. Although it would be better to simulate the state of cells with mutation using point knock-in techniques, this approach carries technical risks. Therefore, we used the CRISPR-KO technique to completely eliminate the presence of FBXW7 in the cell background. FBXW7 mutation in cells was simulated by transfecting FBXW7 mutant plasmids. Among the five mutation forms, we identified that S327X, in which the mutation site was in the first WD40 domain, had an effect similar to FBXW7 knockout, and led to the inactivation of FBXW7 in ESCC cells. The point mutations S382F, D400N and R425C, which are located in the third or fourth WD40 domain, had a weak carcinogenetic function in ESCC. The S598X mutation, which is located outside of the WD40 domain in the C tail of FBXW7, revealed a tiny promotion in ESCC cells. This result indicates that the functional loss of the WD40 domain almost completely inhibits the function of FBXW7, and the point mutation may only weaken its function. Future clinical research should pay more attention to mutations in the WD40 domain than other domains in ESCC.

FBXW7 is one component of the ubiquitin binding system, and many famous oncogenes are the target proteins of FBXW7, such as cyclin E, c-Myc and Notch [31–33]. FBXW7 loss of function leads to upregulation of oncogenes, which in turn promotes tumors [34–36]. The downstream target protein of FBXW7 in ESCC cells needs further study. The present study used GST pulldown and LC–MS/MS analysis for screening, and we found that MAP4 was a potential target of FBXW7. Because the substrates of FBXW7 contain a sequence termed CDPs, we hypothesized that there was a CDP phosphorylated sequence in MAP4. By further screening the phosphorylation database, we finally identified that the threonine T521 of MAP4 played an important role in the FBXW7 degradation system. This amino acid residue is phosphorylated by CHEK1, which was confirmed by the CHEK1 inhibitor CHR-124. Our study found that the recognition substrate motif of FBXW7 target could also be phosphorylated by CHEK1, consistent with others [37]. Further research found that ERK phosphorylation and downstream MMP3 and VEGFA were regulated by FBXW7 via MAP4. In conclusion, we discovered a novel regulatory FBXW7/MAP4/ERK axis which promoting the proliferation, invasion and migration of ESCC cells.

Notably, we found that MAP4 also regulated the expression of FBXW7. The expression of FBXW7 was upregulated after MAP4 inhibition. FBXW7 also elevated following CHEK1 overexpression but decreased after CHIR-124 treatment, which was opposite of MAP4 expression. This regulatory effect may be achieved via ERK phosphorylation. We found FBXW7 expression was increased after ERK inhibitor treatment. And our previous study found that MAP4 interacted with ERK at the protein level and regulated ERK. Several studies showed that ERK phosphorylation inhibited the expression of FXBW7 in cancer [38]. FBXW7 can interact with ERK kinases, resulting in the phosphorylation of FBXW7 at Thr205, which encouraging self-ubiquitination and degradation triggers [39]. These results suggest that ERK plays a central role in the relationship between FBXW7 and MAP4 and promotes the malignant progression of ESCC. We will further explore this mechanism in future studies.

Previous studies and the present study found that the protein level of FBXW7 was reduction, and MAP4 was upregulated in ESCC using immunohistochemistry [9, 19]. The present study further found that the upregulation of MAP4 protein levels was associated with FBXW7 loss of function. Kaplan–Meier (KM) survival curve and multivariate Cox regression analyses revealed a novel biomarker combination and positive expression of FBXW7 with MAP4 weakness that predicted longer survival. However, the RNA level of MAP4 is not the same in the open access database of RNA-seq, which suggests that the abnormality of MAP4 in tumors may be regulated by post-translational modification rather than at the transcriptional level.

There is a strong association between inactivation of FBXW7 and the development of drug resistance in the treatment of tumors [40]. Therefore, it is important to find drug targets for patients with FBXW7 inactivation. As detailed above, activation of ERK pathway have been associated with FBXW7 loss of function. Targeting the aberrant activation of ERK would be an important strategy in ESCC. Notably, when we changed the state of FBXW7, such as knockout or transfection mutants, the inhibitory effect of MK-8353 also changed. MK-8353 exerted better effects when FBXW7 had a loss of function. Besides, bevacizumab is an anti-VEGF monoclonal antibody that is applied in several cancers. Although bevacizumab has not been used alone in ESCC, it has been approved as a combination regimen with PD-1 or chemotherapy drugs. Based on this, we designed a combination drug regimen combined with MK8353 and bevacizumab, founding that the combination drug achieved good results in animals with FBXW7 deletion or mutation. This result suggests that ERK inhibitors are more useful in patients with FBXW7 loss of function in future clinical trials.

## Conclusions

Our current findings disclosed a novel FBXW7/MAP4/ERK axis in esophageal squamous cell carcinoma. Dysregulation of this axis linking functional inactivation mutation and protein disorder may effectively accelerate esophageal squamous cell carcinoma proliferation and provide novel potential biomarkers and targets for esophageal squamous cell carcinoma.

## Supplementary Information

**Additional file 1:** **Supplementary Figure 1.**FBXW7 alteration frequency in different type of cancers in cBioPortal. **Supplementary Figure 2.** Schematic diagram for the mutation sites and sequences of FBXW7. **Supplementary Figure 3.** (A) Quantitation protein expression of FBXW7 relative to KYSE70 in ESCC cell lines. (B) Quantitation proteinexpression of FBXW7 relative to GAPDH in KYSE70 and KYSE180 cells after transfected with sgRNA. Mean ± SEM are shown. ***, *p*<0.001. **Supplementary Figure 4.** Representative images and statistical plots of migration assays in KYSE70 and KYSE180 cells after FBXW7 knockout or rescued with FBXW7 plasmid in gFBXW7-1 cells. Mean ± SEM are shown. ***, *p*<0.001. **Supplementary Figure 5.** Representative images and statistical plots of migration assays in KYSE70 and KYSE180 cells with reversion of different FBXW7 plasmids transfected in FBXW7-KO cells. Mean ± SEM are shown. *, *p*<0.05, ***, *p*<0.001. **Supplementary Figure 6.** (A) Quantitation protein expression of p-ERK, ERK, VEGFA, MMP3 relative to GAPDH in negative control and FBXW7 knockout KYSE70 and KYSE180 cells. (B) Quantitation protein expression of p-ERK, ERK, VEGFA, MMP3 relative to GAPDH in KYSE70 and KYSE180cells in which FBXW7 was knocked out and transfected with different FBXW7 plasmids. (C) Western blot analysis of FBXW7, p-ERK, ERK, VEGFA, MMP3 and GAPDH in KYSE70 and KYSE180 cells treated with DMSO or the ERK inhibitor MK-8353. Means ± SEM are shown. *, *p*<0.05, **, *p*<0.01, ***, *p*<0.001; n.s., not significant. **Supplementary Figure 7.** (A) Quantitation protein expression of MAP4, FBXW7 relative to GAPDH in negative control and FBXW7 knockout KYSE70 and KYSE180 cells. (B) Quantitation protein expression of MAP4 relative to GAPDH in FBXW7-KO cells (gFBXW7-1) transfected with FBXW7 plasmid or EV (empty vector) and treated with cycloheximide (CHX) for different lengths of time. (C) Quantitation protein expression of MAP4 relative to GAPDH in FBXW7-KO cells transfected with FBXW7 plasmid or EV and treated with DMSO or the proteasomal inhibitor MG132. (D) Quantitation protein expression of MAP4 relative to GAPDH in KYSE70 and KYSE180 cells transfected with different MAP4 plasmids. (E) Quantitation protein expression of MAP4 relative to FBXW7 input in the immunoprecipitation analysis of the interaction between FBXW7 and different MAP4 plasmids in KYSE70 cells. (F) Quantitation protein expression of p-ERK, ERK, VEGFA, MMP3, FBXW7, MAP4 relative to GAPDH in the negative control, CHEK1-overexpressing KYSE70 and KYSE180 cells treated with the CHEK1 inhibitor CHIR-124. (G) Quantitation protein expression of MAP4, p-ERK, ERK, MMP3, VEGFA, FBXW7 relative to GAPDH in negative control and MAP4 knockdown KYSE70 and KYSE180 cells. Means ± SEM are shown. *, *p*<0.05,**, *p*<0.01, ***, *p*<0.001; n.s., not significant. **Supplementary Figure 8.** (A) The RNA-seq FPKM of FBXW7 in TCGA ESCC database. (B) The RNA-seq FPKM of MAP4 in TCGA ESCC database. n.s., not significant. **Supplementary Figure 9.** (A) The mRNA expression correlation between FBXW7 and MAP4 in GSE53622 RNA-seq data. (B) The mRNA expression correlation between FBXW7 and MAP4 in GSE53624 RNA-seq data. (C) The mRNA expression correlation between FBXW7 and MAP4 in TCGA RNA-seq data. **Supplementary Figure 10.** The intensity of PCNA staining score in the indicated groups. Mean ± SEM are shown. ***, *p*<0.001; n.s., not significant.

**Additional file 2. **The sgRNA sequences. Primers sequences for plasmid construction. Primer sequences for mutation sequencing. Primer sequences for the quantitative RT-PCR. Relationship between protein overexpression and clinicopathologic parameters in tissues. Relationship between protein overexpression and clinicopathologic parameters in tissues. The relationship between FBXW7 and MAP4 expression in ESCC tissues. Relationship between protein overexpression and clinicopathologic parameters in tissues. Prognostic significance from cox proportional hazards model.  

## Data Availability

The datasets (TCGA.ESCA) analyzed during the current study are available in the UCSC Xena TCGA hub repository, https://tcga.xenahubs.net. The datasets (GSE53622 and GSE53624) analyzed during the current study are available in the GEO database, https://www.ncbi.nlm.nih.gov/geo/.

## Abbreviations

CRISPR: Clustered regularly interspaced short palindromic repeats
siRNA: Small interfering RNA
qPCR: Quantitative polymerase chain reaction
FBXW7: F-box/WD repeat-containing protein 7
MAP4: Microtubule-associated protein 4
ERK: Extracellular signal-regulated kinase 1/2
MMP3: Matrix metallopeptidase 3
VEGFA: Vascular endothelial growth factor A
CHEK1: Checkpoint kinase 1
GST: Glutathione S-transferase
