# Supplementary material for: FBXW7 loss of function promotes esophageal squamous cell carcinoma progression via elevating MAP4 and ERK phosphorylation
Source: J Exp Clin Cancer Res. 2023 Mar 29;42:75. doi: 10.1186/s13046-023-02630-3 (PMC10054043; doi:10.1186/s13046-023-02630-3)
Supplement: Supplementary file 1 — Additional file 1: Supplementary Figure 1.FBXW7 alteration frequency in different type of cancers in cBioPortal. Supplementary Figure 2. Schematic diagram for the mutation sites and sequences of FBXW7. Supplementary Figure 3. (A) Quantitation protein expression of FBXW7 relative to KYSE70 in ESCC cell lines. (B) Quantitation proteinexpression of FBXW7 relative to GAPDH in KYSE70 and KYSE180 cells after transfected with sgRNA. Mean ± SEM are shown. ***, p<0.001. Supplementary Figure 4. Representative images and statistical plots of migration assays in KYSE70 and KYSE180 cells after FBXW7 knockout or rescued with FBXW7 plasmid in gFBXW7-1 cells. Mean ± SEM are shown. ***, p<0.001. Supplementary Figure 5. Representative images and statistical plots of migration assays in KYSE70 and KYSE180 cells with reversion of different FBXW7 plasmids transfected in FBXW7-KO cells. Mean ± SEM are shown. *, p<0.05, ***, p<0.001. Supplementary Figure 6. (A) Quantitation protein expression of p-ERK, ERK, VEGFA, MMP3 relative to GAPDH in negative control and FBXW7 knockout KYSE70 and KYSE180 cells. (B) Quantitation protein expression of p-ERK, ERK, VEGFA, MMP3 relative to GAPDH in KYSE70 and KYSE180cells in which FBXW7 was knocked out and transfected with different FBXW7 plasmids. (C) Western blot analysis of FBXW7, p-ERK, ERK, VEGFA, MMP3 and GAPDH in KYSE70 and KYSE180 cells treated with DMSO or the ERK inhibitor MK-8353. Means ± SEM are shown. *, p<0.05, **, p<0.01, ***, p<0.001; n.s., not significant. Supplementary Figure 7. (A) Quantitation protein expression of MAP4, FBXW7 relative to GAPDH in negative control and FBXW7 knockout KYSE70 and KYSE180 cells. (B) Quantitation protein expression of MAP4 relative to GAPDH in FBXW7-KO cells (gFBXW7-1) transfected with FBXW7 plasmid or EV (empty vector) and treated with cycloheximide (CHX) for different lengths of time. (C) Quantitation protein expression of MAP4 relative to GAPDH in FBXW7-KO cells transfected with FBXW7 plasmid or EV and [file 13046_2023_2630_MOESM1_ESM.docx]

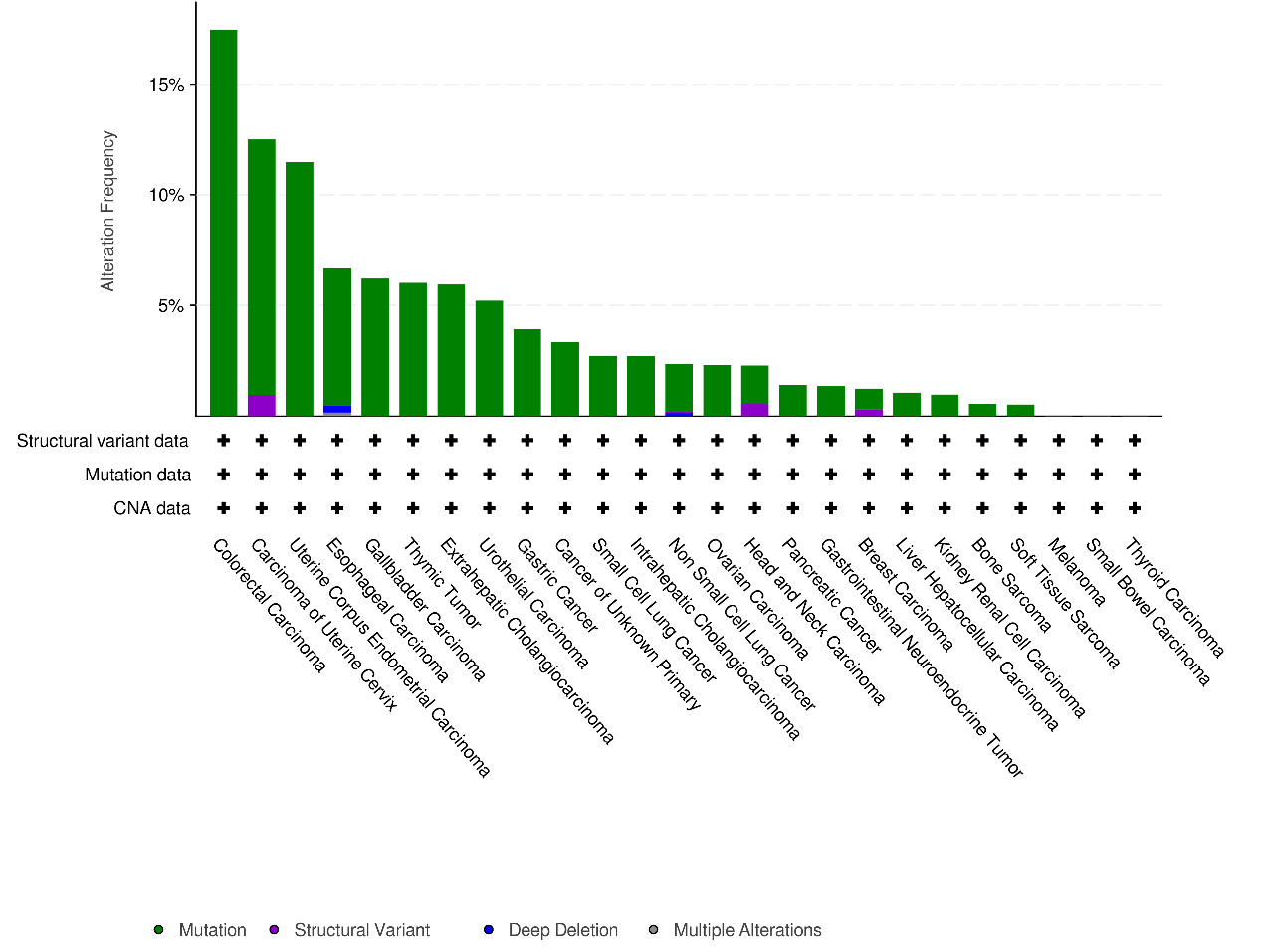


Supplementary Figure 1

FBXW7 alteration frequency in different type of cancers in cBioPortal.


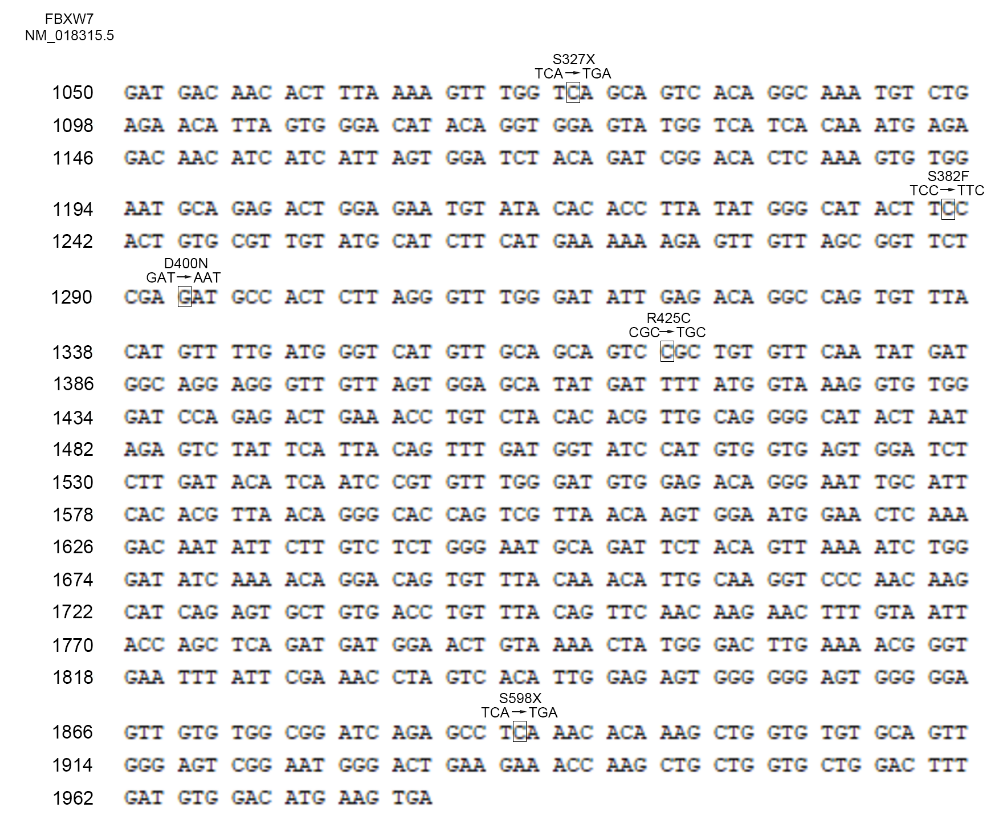


Supplementary Figure 2

Schematic diagram for the mutation sites and sequences of FBXW7


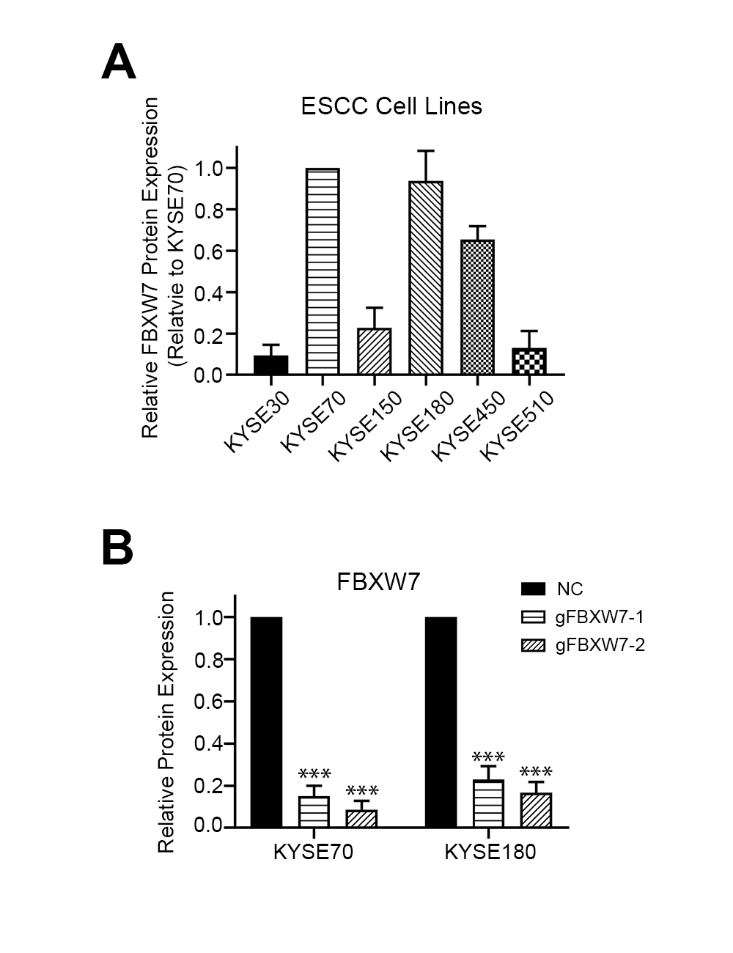


Supplementary Figure 3

1. Quantitation protein expression of FBXW7 relative to KYSE70 in ESCC cell lines.
2. Quantitation protein expression of FBXW7 relative to GAPDH in KYSE70 and KYSE180 cells after sgRNA and transfected with plasmid in gFBXW7-1 cells.

Mean ± SEM are shown. ***, p<0.001.


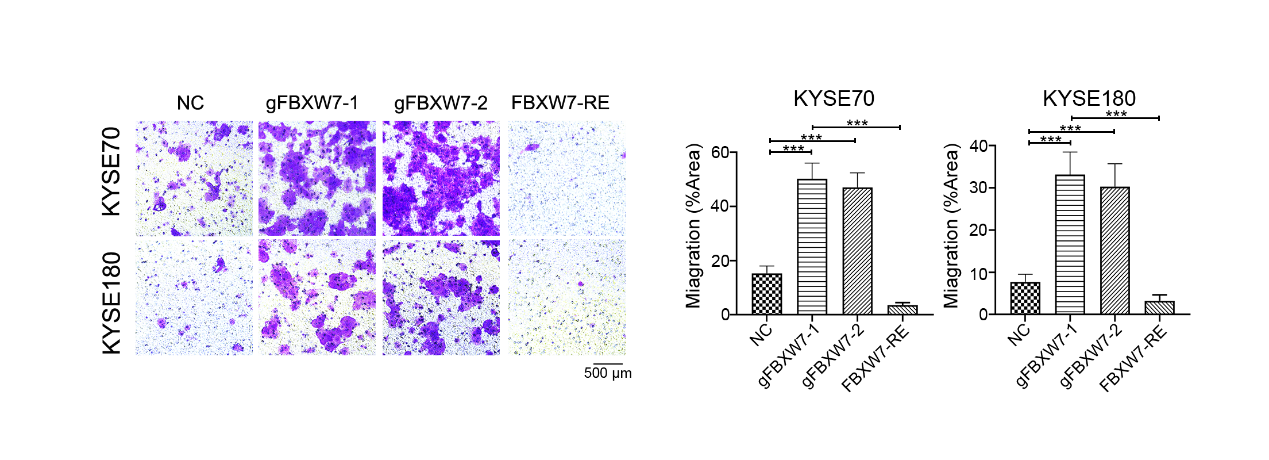


Supplementary Figure 4

Representative images and statistical plots of migration assays in KYSE70 and KYSE180 cells after FBXW7 knockout or rescued with FBXW7 plasmid in gFBXW7-1 cells. Mean ± SEM are shown. ***, p<0.001.


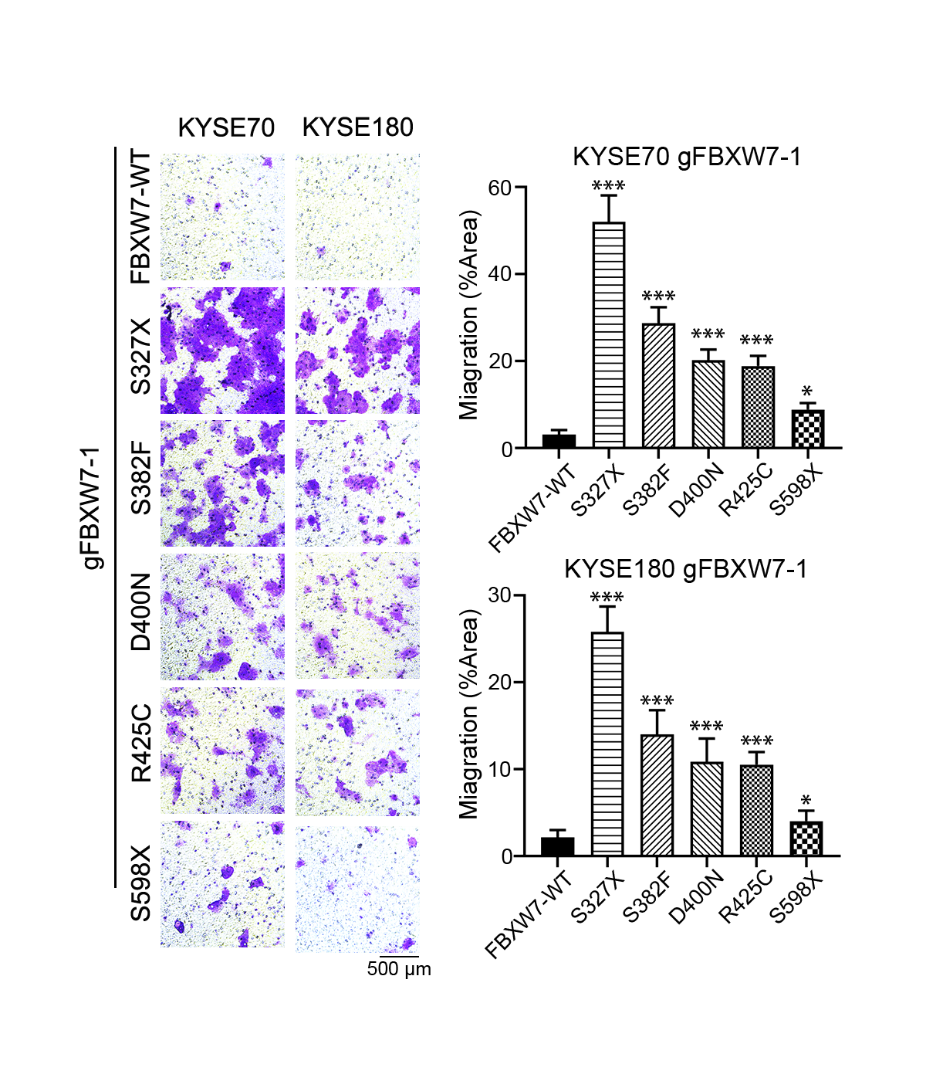


Supplementary Figure 5

Representative images and statistical plots of migration assays in KYSE70 and KYSE180 cells with reversion of different FBXW7 plasmids transfected in FBXW7-KO cells. Mean ± SEM are shown. *, p<0.05, ***, p<0.001.


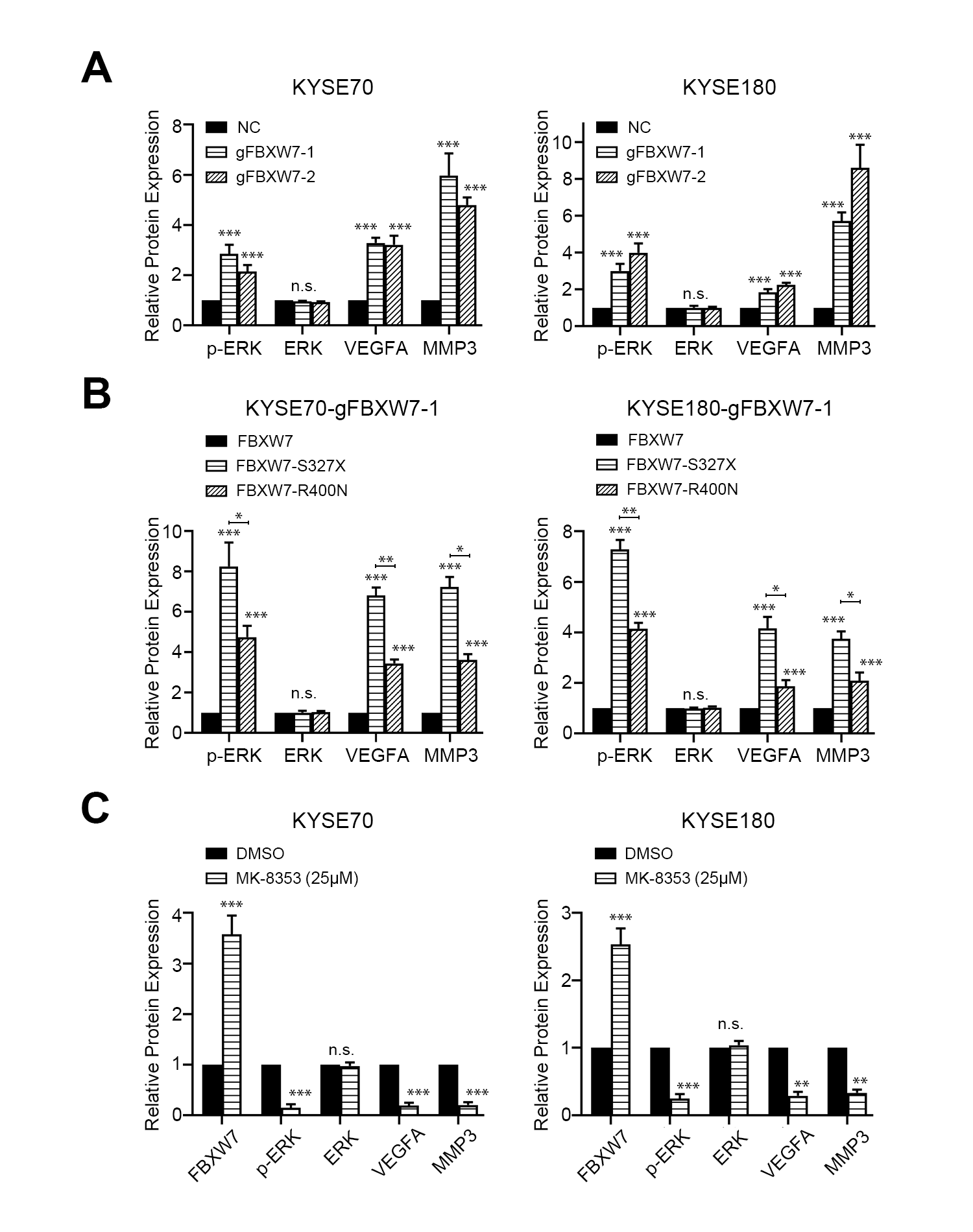


Supplementary Figure 6

1. Quantitation protein expression of p-ERK, ERK, VEGFA, MMP3 relative to GAPDH in negative control and FBXW7 knockout KYSE70 and KYSE180 cells.
2. Quantitation protein expression of p-ERK, ERK, VEGFA, MMP3 relative to GAPDH in KYSE70 and KYSE180 cells in which FBXW7 was knocked out and transfected with different FBXW7 plasmids.
3. Western blot analysis of FBXW7, p-ERK, ERK, VEGFA, MMP3 and GAPDH in KYSE70 and KYSE180 cells treated with DMSO or the ERK inhibitor MK-8353.

Means ± SEM are shown. *, p<0.05, **, p<0.01, ***, p<0.001; n.s., not significant.


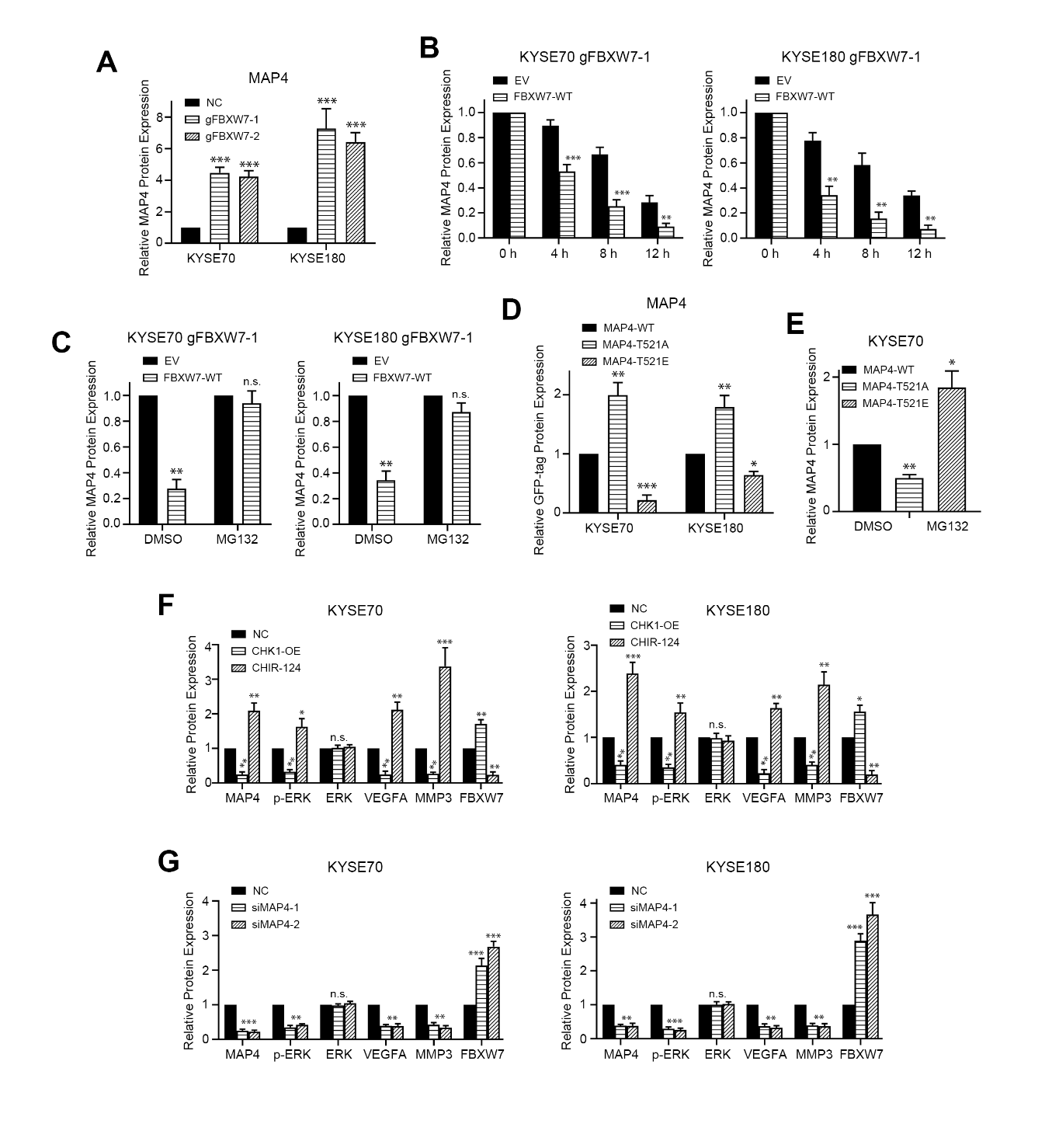


Supplementary Figure 7

1. Quantitation protein expression of MAP4, FBXW7 relative to GAPDH in negative control and FBXW7 knockout KYSE70 and KYSE180 cells.
2. Quantitation protein expression of MAP4 relative to GAPDH in FBXW7-KO cells (gFBXW7-1) transfected with FBXW7 plasmid or EV (empty vector) and treated with cycloheximide (CHX) for different lengths of time.
3. Quantitation protein expression of MAP4 relative to GAPDH in FBXW7-KO cells transfected with FBXW7 plasmid or EV and treated with DMSO or the proteasomal inhibitor MG132.
4. Quantitation protein expression of MAP4 relative to GAPDH in KYSE70 and KYSE180 cells transfected with different MAP4 plasmids.
5. Quantitation protein expression of MAP4 relative to FBXW7 input in the immunoprecipitation analysis of the interaction between FBXW7 and different MAP4 plasmids in KYSE70 cells.
6. Quantitation protein expression of p-ERK, ERK, VEGFA, MMP3, FBXW7, MAP4 relative to GAPDH in the negative control, CHK1-overexpressing KYSE70 and KYSE180 cells treated with the CHK1 inhibitor CHIR-124.
7. Quantitation protein expression of MAP4, p-ERK, ERK, MMP3, VEGFA, FBXW7 relative to GAPDH in negative control and MAP4 knockdown KYSE70 and KYSE180 cells.

Means ± SEM are shown. *, p<0.05, **, p<0.01, ***, p<0.001; n.s., not significant.


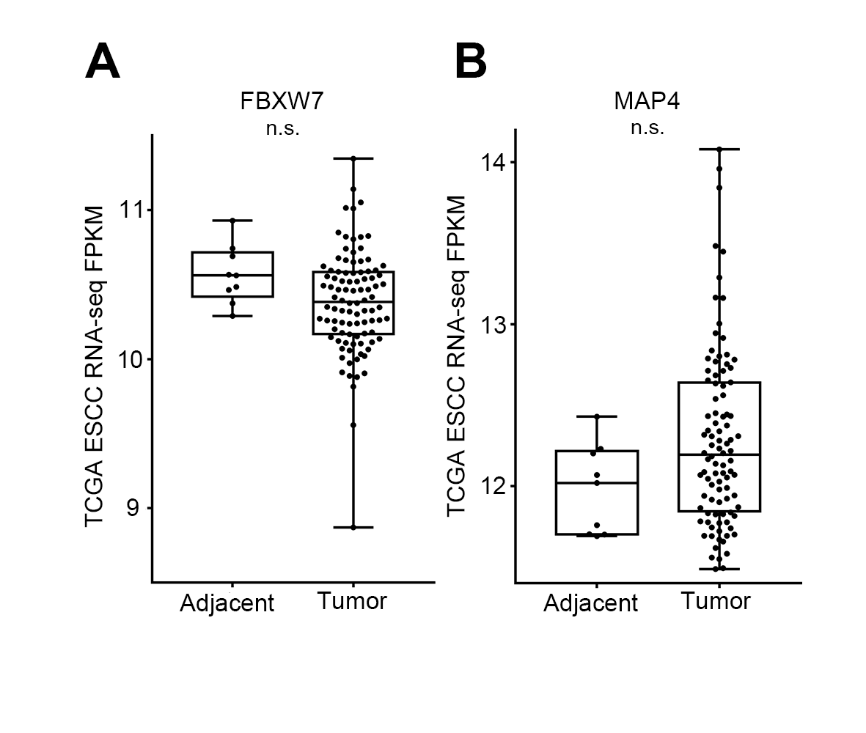


Supplementary Figure 8

1. The RNA-seq FPKM of FBXW7 in TCGA ESCC database.
2. The RNA-seq FPKM of MAP4 in TCGA ESCC database. n.s., not significant.


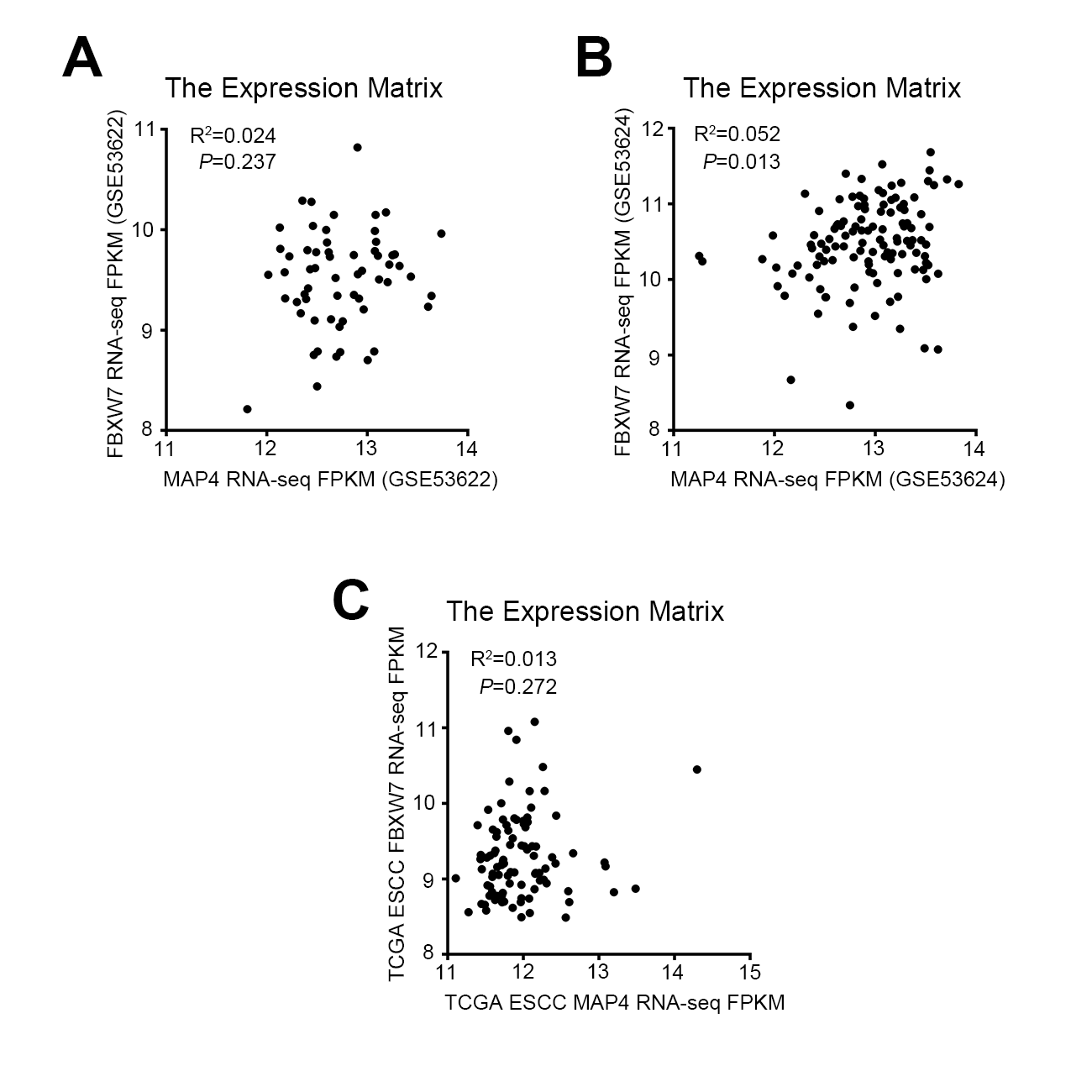


Supplementary Figure 9

1. The mRNA expression correlation between FBXW7 and MAP4 in GSE53622 RNA-seq data.
2. The mRNA expression correlation between FBXW7 and MAP4 in GSE53624 RNA-seq data.
3. The mRNA expression correlation between FBXW7 and MAP4 in TCGA RNA-seq data.


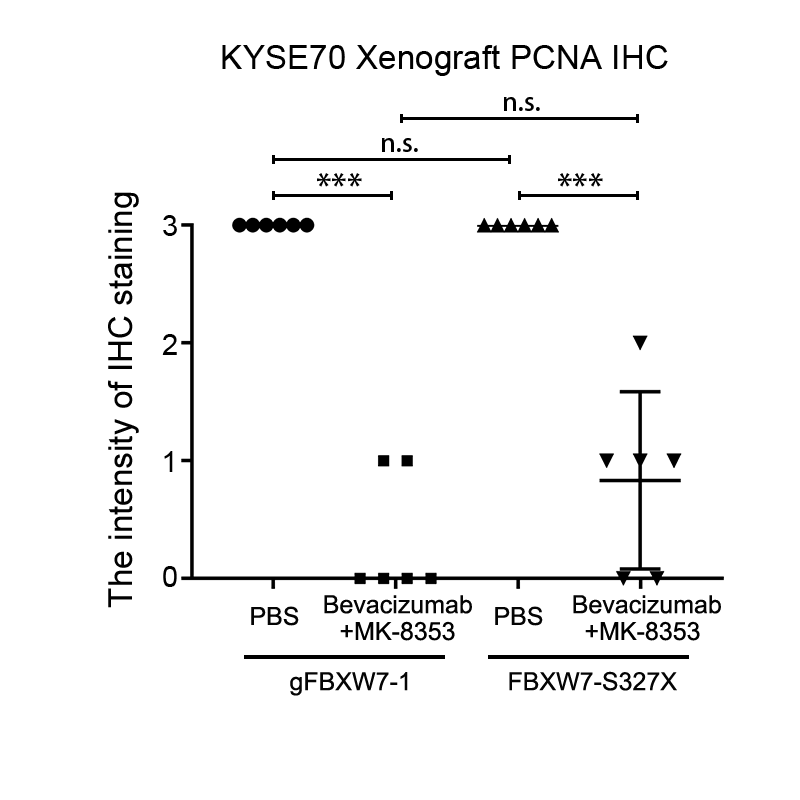


Supplementary Figure 10

The intensity of PCNA staining score in the indicated groups. Mean ± SEM are shown. ***, p<0.001; n.s., not significant.
